# Single‐cell transcriptomic analysis of glioblastoma reveals pericytes contributing to the blood–brain–tumor barrier and tumor progression

**DOI:** 10.1002/mco2.70014

**Published:** 2024-12-04

**Authors:** Yuzhe Li, Changwu Wu, Xinmiao Long, Xiangyu Wang, Wei Gao, Kun Deng, Bo Xie, Sen Zhang, Minghua Wu, Qing Liu

**Affiliations:** ^1^ Department of Neurosurgery Xiangya Hospital Central South University Changsha Hunan China; ^2^ Department of Neurosurgery Chinese Academy of Medical Sciences and Peking Union Medical College Beijing China; ^3^ National Clinical Research Center for Geriatric Disorders Xiangya Hospital Central South University Changsha Hunan China; ^4^ Cancer Research Institute Central South University Changsha Hunan China

**Keywords:** blood–brain–tumor barrier, glioblastoma, pericyte, prognosis

## Abstract

The blood–brain barrier is often altered in glioblastoma (GBM) creating a blood–brain–tumor barrier (BBTB) composed of pericytes. The BBTB affects chemotherapy efficacy. However, the expression signatures of BBTB‐associated pericytes remain unclear. We aimed to identify BBTB‐associated pericytes in single‐cell RNA sequencing data of GBM using pericyte markers, a normal brain pericyte expression signature, and functional enrichment. We identified parathyroid hormone receptor‐1 (PTH1R) as a potential marker of pericytes associated with BBTB function. These pericytes interact with other cells in GBM mainly through extracellular matrix–integrin signaling pathways. Compared with normal pericytes, pericytes in GBM exhibited upregulation of several ECM genes (including collagen IV and *FN1*), and high expression levels of these genes were associated with a poor prognosis. Cell line experiments showed that PTH1R knockdown in pericytes increased collagen IV and FN1 expression levels. In mice models, the expression levels of PTH1R, collagen IV, and FN1 were consistent with these trends. Evans Blue leakage and IgG detection in the brain tissue suggested a negative correlation between PTH1R expression levels and blood–brain barrier function. Further, a risk model based on differentially expressed genes in PTH1R^+^ pericytes had predictive value for GBM, as validated using independent and in‐house cohorts.

## Graphical abstract

This study identified a type of pericyte highly related to BBTB function in GBM, with PTH1R potentially serving as a marker for these pericytes. PTH1R^+^ pericytes expressed extracellular matrix (ECM) genes to interact with integrins of other GBM cells, and had increased expression of ECM genes such collagen IV and FN1 in GBM. The expression level of PTH1R was negatively correlated with collagen IV expression, FN1 expression, and BBB function in brain.

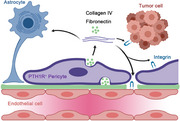

## 1 INTRODUCTION

Glioblastoma (GBM) is the most common malignant brain tumor.^1^ It is characterized by high resistance to postoperative therapy, as reflected in poor prognosis and a high recurrence rate.^2^ The poor prognosis of patients with GBM is attributed to the lack of effective chemotherapeutic agents and unsuccessful drug delivery across the blood–brain barrier (BBB).^3^

The BBB is a cellular barrier comprising specialized brain endothelial cells (ECs), pericytes, astrocytes, and perivascular macrophages.^4^ Moreover, the BBB primarily regulates the passage of substances from the blood to the brain to maintain optimal neuronal activity. However, it restricts the passage of most drug compounds, except for highly lipid‐soluble small molecules with a molecular weight less than 400 Da. Consequently, many anticancer drugs are ineffective at treating GBM.^5^, ^6^ Studies using magnetic resonance and positron emission tomography imaging have shown that a considerable proportion of GBM tissue has a functional BBB.^7^, ^8^ Studies on brain metastases have shown that highly effective targeted therapies that are BBB impenetrable have “brain‐only failures.”^9^ Abnormal vessel formation and disruption of the BBB occur in GBM, stimulated by the increased metabolic demands of GBM cells.^10^, ^11^ This results in a blood–brain–tumor barrier (BBTB). The BBTB hinders the delivery of sufficient therapeutic drugs into GBM tissue due to aberrant expression of transcytotic proteins in tumor vessels.^12^ Thus, overcoming the BBTB is important when treating GBM.

Pericytes are defined by their anatomical location surrounding the walls of blood vessels and they constitute an important component of the BBB.^13^, ^14^ Pericytes communicate with ECs through physical contact via their long processes that surround the blood vessel walls and via paracrine signaling.^15^, ^16^ The coverage ratio of pericytes to ECs is 1/3 in the brain,^17^, ^18^ underscoring the crucial role of pericytes in BBB function. High pericyte coverage in GBM is associated with poor chemotherapy outcomes.^19^ Pericytes in different locations provide structural support to the microvasculature and play important roles in blood vessel formation,^20^ blood flow regulation, blood vessel permeability, immune function,^21^ coagulation,^22^ pinocytosis/phagocytosis,^23^ and communication with ECs and astrocytes. Pericytes express a wide variety of markers and originate from different sources.^24^ Recent studies have revealed that pericytes exhibit significant differences between each other in both their marker expression and function.^25^, ^26^ Even recognized pericyte markers, such as αSMA, NG2, PDGFRβ, CD13, and desmin, have been found to be expressed in other cell types. NG2 is also expressed in oligodendrocyte precursors and microglia.^27^, ^28^ PDGFRβ is expressed in smooth muscle cells,^29^ fibroblasts,^30^ and mesenchymal stem cells.^31^ Thus, these markers are not specific for pericyte identification. Capillary pericytes are negative for αSMA and positive for desmin, whereas venular pericytes express both markers.^32^ Identifying more specific markers for pericytes with distinct functions is necessary to better understand the complex perivascular microenvironment.

It has been observed that tumors with lower pericyte coverage rates are more susceptible to radiation and chemotherapy.^33^, ^34^ Research by Cheng et al.^35^ showed that most pericytes in GBM are derived from GBM stem cells, indicating that pericytes in GBM may have distinct markers and functions. Identifying these markers and functions may reveal novel therapeutic options for BBTB. The objective of this study was to identify pericytes that contribute to BBTB function in GBM and investigate their impact on the prognosis of patients with GBM.

In this study we identified a type of pericyte highly related to BBTB function in GBM, with parathyroid hormone (PTH)1R potentially serving as a marker for these pericytes. Then we explored the mechanism of how this pericytes affect BBTB function in tumor. This study offers new perspectives for exploring BBTB function and the GBM tumor microenvironment.

## 2 RESULTS

### 2.1 Identification of pericytes involved in BTBB function in GBM

**FIGURE 1:**
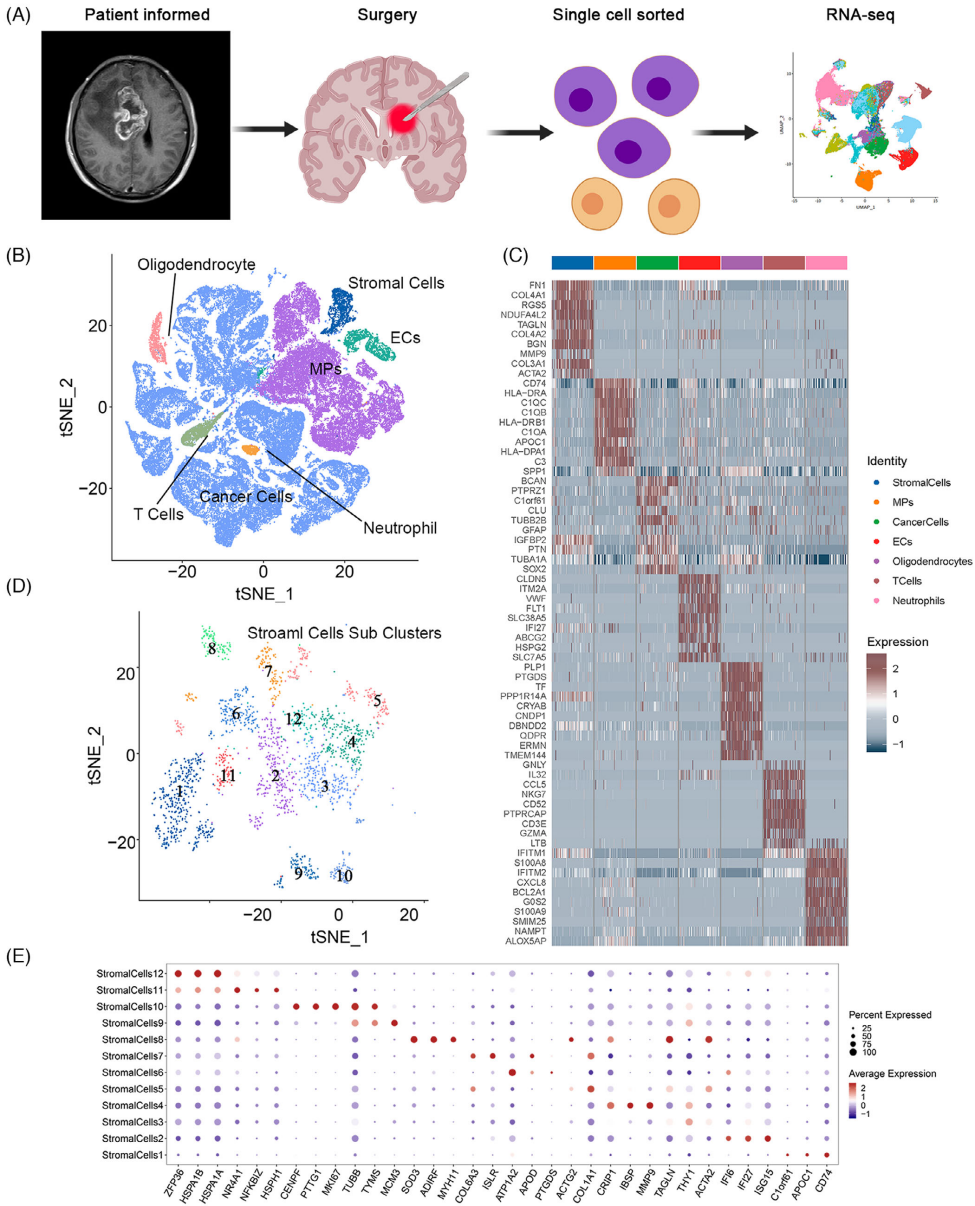
Overview of integrated scRNA‐seq analysis of nine glioblastoma samples. (A) Schematic overview of the single‐cell RNA sequencing (scRNA‐seq) analysis protocol. (B) A total of 93,343 cells are visualized in the t‐distributed stochastic neighbor embedding (t‐SNE) plot. (C) Differentially expressed genes (DEGs) of all clusters shown in the heatmap. (D) Subclusters of stromal cells visualized in the t‐SNE plot. (E) DEGs of stromal cell subclusters shown in the bubble chart.

**FIGURE 2:**
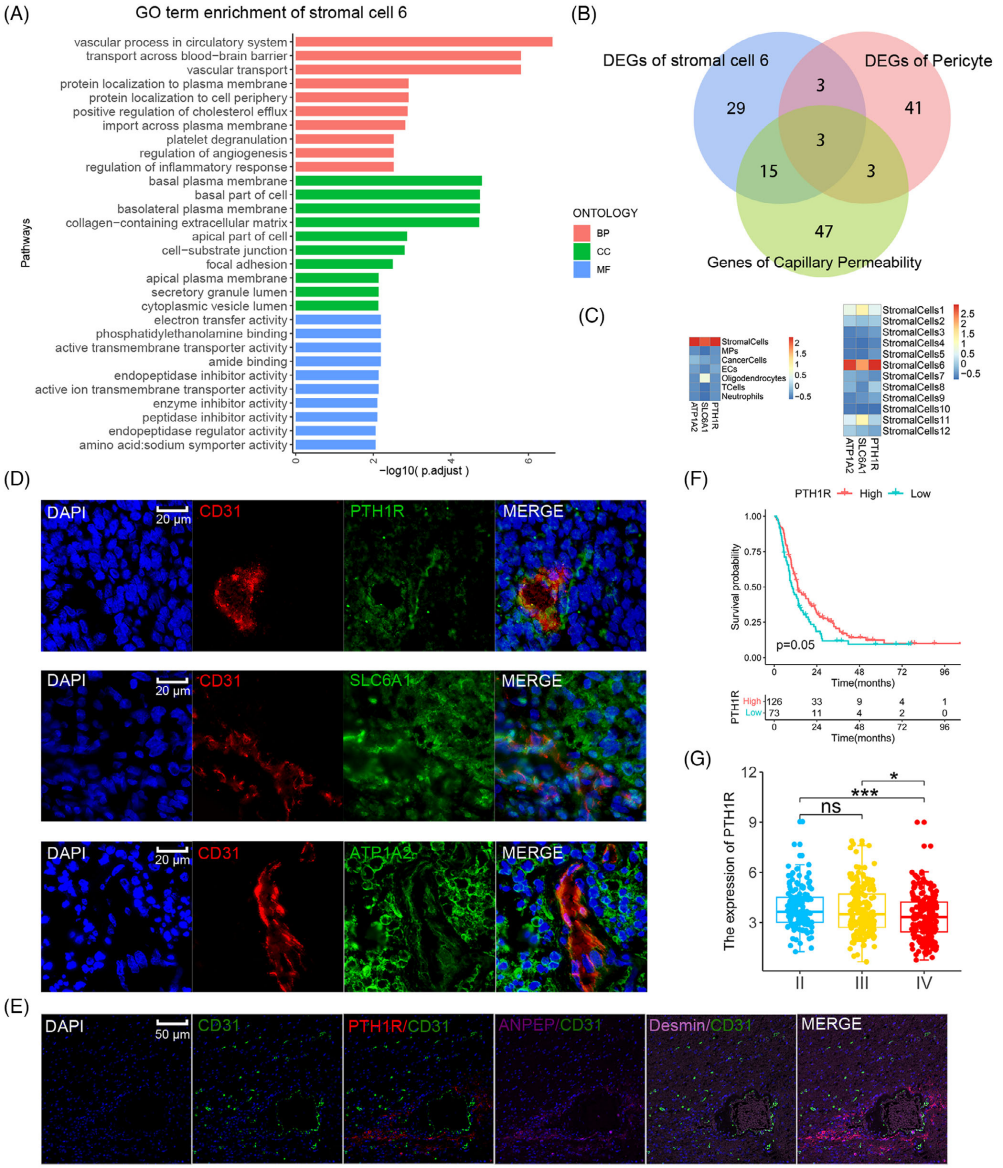
Verification of markers of pericytes associated with blood–tumor–brain barrier function. (A) Gene Ontology (GO) enrichment analysis of cluster 6. (B) Intersection of DEGs in stromal cell cluster 6, DEGs in brain pericytes, and genes from GO terms involving blood–brain barrier (BBB) function. (C) Expression of overlapping genes in all general clusters and stromal cell subclusters. (D) Costaining of potential pericyte hallmarks and CD31. CD31ias marked red and the target genes are marked green. (E) Multi‐immunofluorescence costaining of CD31, PTH1R, ANPEP, and desmin. Red, PTH1R; green, CD31; rose, ANPEP; pink, desmin. The results of PTH1R, ANPEP, and desmin costaining with CD31 are shown to compare the specificity of the three markers. (F) Survival curve between the high‐ and low‐PTH1R‐expression groups in the CGGA GBM cohort. G PTH1R expression levels according to glioma grade in the CGGA cohort.

Single‐cell RNA sequencing (scRNA‐seq) analysis was applied to identify the pericytes in GBM. Nine fresh GBM tumor samples were selected (Table S1), and 93,343 cells passed quality control and were included in this study. Graph‐based clustering analysis, using known lineage markers, identified seven cell populations, and their characteristics are shown on the heatmap in Figure 1B,C. A highly heterogeneous population of cancer cells was observed due to GBM cell heterogeneity among samples (Figure S1). Pericytes are a type of stromal cell. Stromal cells comprised 2.38% of the total cell count in the nine specimens, which including 2217 cells. Further clustering divided the stromal cells into 12 subclusters, with their markers shown in the bubble diagram. Differentially expressed genes (DEGs) were identified by comparing each stromal cell cluster with other stromal cells (Figure 1D,E). The results indicated that pericyte markers from previous studies displayed discrete distributions within stromal cell subclusters (Figure S2), suggesting significant heterogeneity in pericyte definitions among previous studies (Table 1). A previous snRNA‐seq study identified two types of pericytes in normal brain tissue: arterial‐smooth‐muscle‐cell‐like pericytes and venous‐smooth‐muscle‐cell‐like pericytes.^36^ The 50 most highly upregulated DEGs of these pericytes (DEGs compared with other vascular cells) were considered as the normal brain pericyte expression signature and were used in the gene set variation analysis (GSVA) and gene set enrichment analysis (GSEA) of stromal cell subclusters. Clusters 1, 6, and 11 were enriched in the pericyte expression signature in GSVA and GSEA (Figure S2). Clusters 1, 6, and 11 also expressed the well‐recognized pericyte marker, PDGFRβ, with cluster 11 showing the highest level of enrichment. Therefore, stromal cell subclusters 1, 6, and 11 were annotated as pericytes in GBM. DEGs between each stromal cell cluster and other stromal cells were used in Gene Ontology (GO) term analysis. Different biological functions of pericytes, such as contraction, cell adhesion, immune functions, and vascular processes, were found in distinct clusters (Figure S3). Cluster 6 was characterized by the following biological process terms related to vascular function and permeability: “vascular process in circulatory system,” “transport across the blood–brain barrier,” and “vascular transport.” The cellular component terms in this cluster pertained to basal plasma membrane and cell adhesion, as follows: “basal plasma membrane,” “basolateral plasma membrane,” “collagen‐containing extracellular matrix,” “cell‐substrate junction,” and “focal adhesion.” The molecular function terms in cluster 6 were associated with transmembrane transporter activities (Figure 2A). The GO functional enrichment analysis demonstrated significantly enriched functions relating to the structure and permeability of the BBB in cluster 6, indicating a close association between cluster 6 and BTBB functions in GBM. Additionally, clusters 1 and 11 were enriched in pathways relating to cellular adhesion, biosynthesis, and the regulation of cellular morphology; however, vascular function and permeability were not enriched, indicating that these pericytes did not play a major role in BBTB function. Of note, cluster 8 expressed a specific marker of mesenchymal stem cells (CD44) and highly expressed most pericyte markers (DES, αSMA, CALD1, and Ang1). Furthermore, the GSVA findings for pericyte expression signatures were insignificant (Figure S2). The conflicting findings from GSVA, GO functional enrichment analysis, and biomarker expression suggested that the identification of pericytes requires multiple approaches.

**TABLE 1:** Expression of pericyte markers in previous studies of stromal cell sub clusters.

Pericyte markers	Cross expression	Enrichment	References
αSMA (ACTA2)	Smooth muscle cells	Cluster 6	^25^
NG2 (CAPG4)	Oligodendrocyte progenitors	Cluster 11	^84^
PDGFRβ	Fibroblasts	Cluster 11	^30^
Kir6.1 (KCNJ8)	Smooth muscle cells	Cluster 6	^85^
VIM	Endothelial cells	Cluster 5	^86^
MYH11	Only arteriolar pericytes	Cluster 8	^26^
RGS5	Smooth muscle cells	Cluster 12	^87^
ABCC1	Smooth muscle cells	Cluster 11	^25^
ABCC9	Smooth muscle cells	/	^85^
ALPL	Endothelial cells	Cluster 5	^88^
MCAM (CD146)	Mesenchymal stem cells	/	^89^
PROM1	Glioma stem cells	Cluster 1	^90^
Glast (SLC1A3)	Only spinal cord pericytes	Cluster 6	^91^
LEPR	Sinusoidal pericytes	Cluster 8	^92^
Nestin (NES)	Only type‐2 pericytes	/	^92^, ^93^
Gli1	Several perivascular cells	/	^94^
PDGFRa	Fibroblasts	Cluster 1, 7	^91^
TBX18	Smooth muscle cells	Cluster 8	^25^
DLK1	Smooth muscle cells	Cluster 1	^85^
Endosialin (CD248)	Fibroblasts and T cells	/	^95^
CD13 (ANPEP)	Smooth muscle/endothelial cells	Cluster 6	^96^
CD44	Lung cancer stem cells	Cluster 8	^97^
THY1	/	/	^98^
ANGTP1	Endothelial cells	Cluster 8	^99^
CALD1	Endothelial cells	Cluster 8	^100^
BMX	/	/	^101^

In addition, trajectory analysis using Monocle2 indicated that the majority of the three pericyte clusters (clusters 1, 6, and 11) differentiated along the same branch, with cluster 1 being the terminal of the branch and cluster 8 (mesenchymal stem cells) being the origin. RNA velocity analysis suggested that cluster 6 was derived from mesenchymal stem cells (cluster 8) (Figure S4). The abovementioned results demonstrated that stromal cell subcluster 6 comprised pericytes with BTBB functions.

### 2.2 Pertinent marker for pericytes with BTBB function

To determine potential markers related to BBTB function, the intersection of the 50 most highly upregulated DEGs in pericytes with BBTB functions (compared with other stromal cells in GBM), normal brain pericyte (venous‐smooth‐muscle‐cell‐like pericytes) expression signatures, and genes associated with GO terms related to capillary permeability was used. After this intersection analysis, three genes remained: *ATP1A2*, *SLC6A1*, and *PTH1R* (Figure 2B). These three genes had high specificity for stromal cell cluster 6 (Figure 2C). The results of CD31 (marking ECs) costaining indicated that cells positive for PTH1R exhibited morphological cohesion with the blood vessels of GBM tissue, exhibiting morphological characteristics identical to those of pericytes (Figure 2D). Immunofluorescence (IF) staining of PTH1R, CD31, and recognized pericytes markers (ANPEP and desmin) was performed in GBM tissue to compare the specificity of PTH1R with that of other markers of pericytes. The colocalization results demonstrated that PTH1R had a similar specificity to that of ANPEP. PTH1R and ANPEP both had a significantly higher specificity compared with that of desmin (Figure 2E). A significant negative correlation was observed between higher expression levels of PTH1R and prognosis in the CGGA GBM cohort (Figure 2F) and the expression level of PTH1R was significantly lower in high‐grade GBM than low‐grade GBM (Figure 2G). These findings indicate that PTH1R can be regarded as a specific marker of pericytes associated with BTBB function in GBM.

### 2.3 PTH1R^+^ pericytes communicate with other cells via extracellular matrix–integrin signaling pathways

**FIGURE 3:**
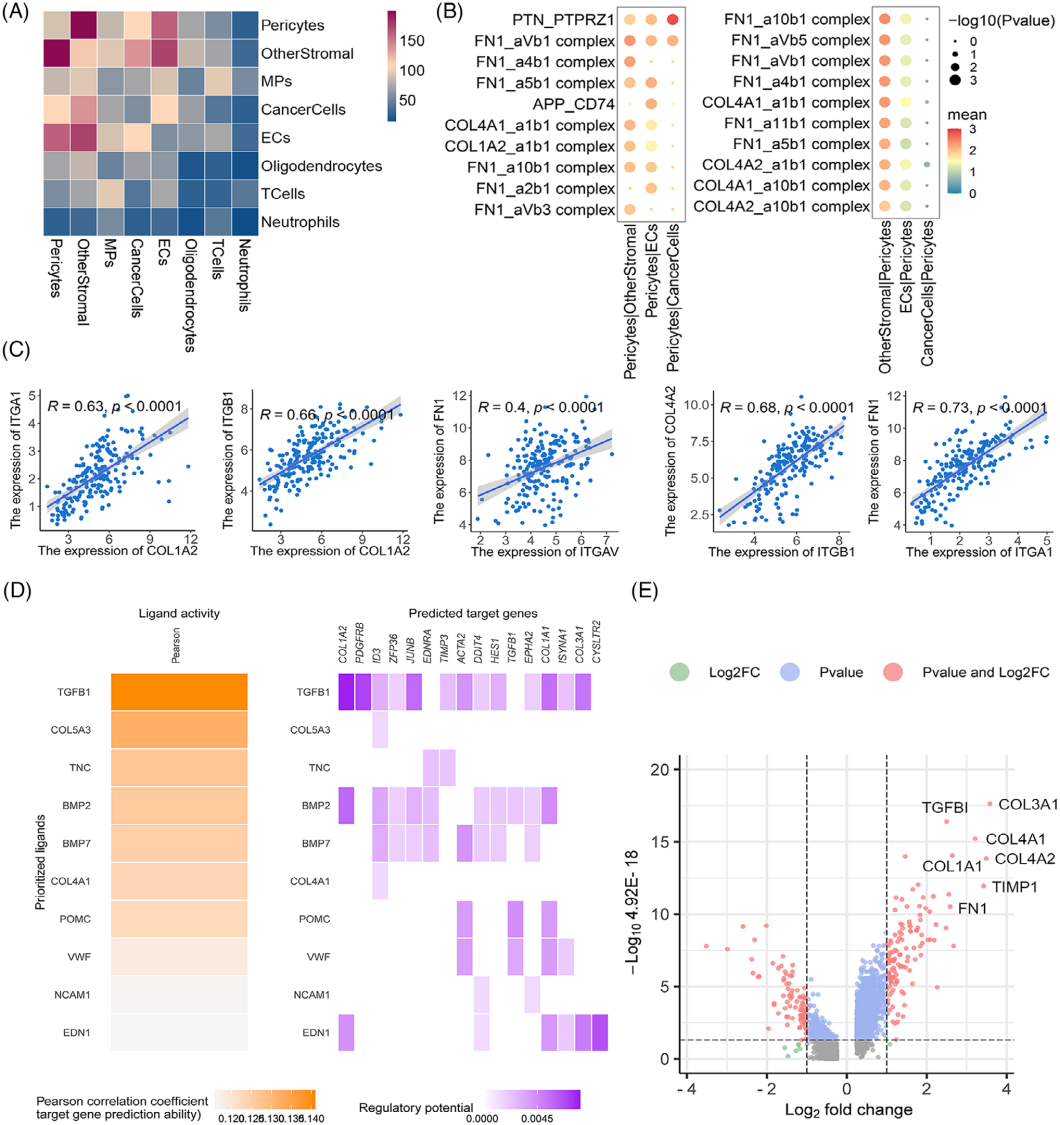
PTH1R+ pericytes communicate via extracellular‐matrix‐integrin signaling pathways. (A) PTH1R^+^ pericytes interact with other cells, as determined using CellPhoneDB analysis. (B) Significant interaction pair between PTH1R^+^ pericytes and cells of interest in the CellPhoneDB analysis. (C) Correlations between interaction pairs in the CGGA cohort. (D) PTH1R+ pericytes interact with other cells, as determined using NicheNet analysis. DEGs between pericytes associated with blood–tumor–brain barrier (BBTB) function and other stromal cells were defined as the gene set of interest. All other genes expressed in the receiver tissue (average mean expression over all conditions > 1 tag/kb) were considered as background. (E) DEGs between pericytes in GBM and normal brain tissue in the GSE162631 dataset.

**FIGURE 4:**
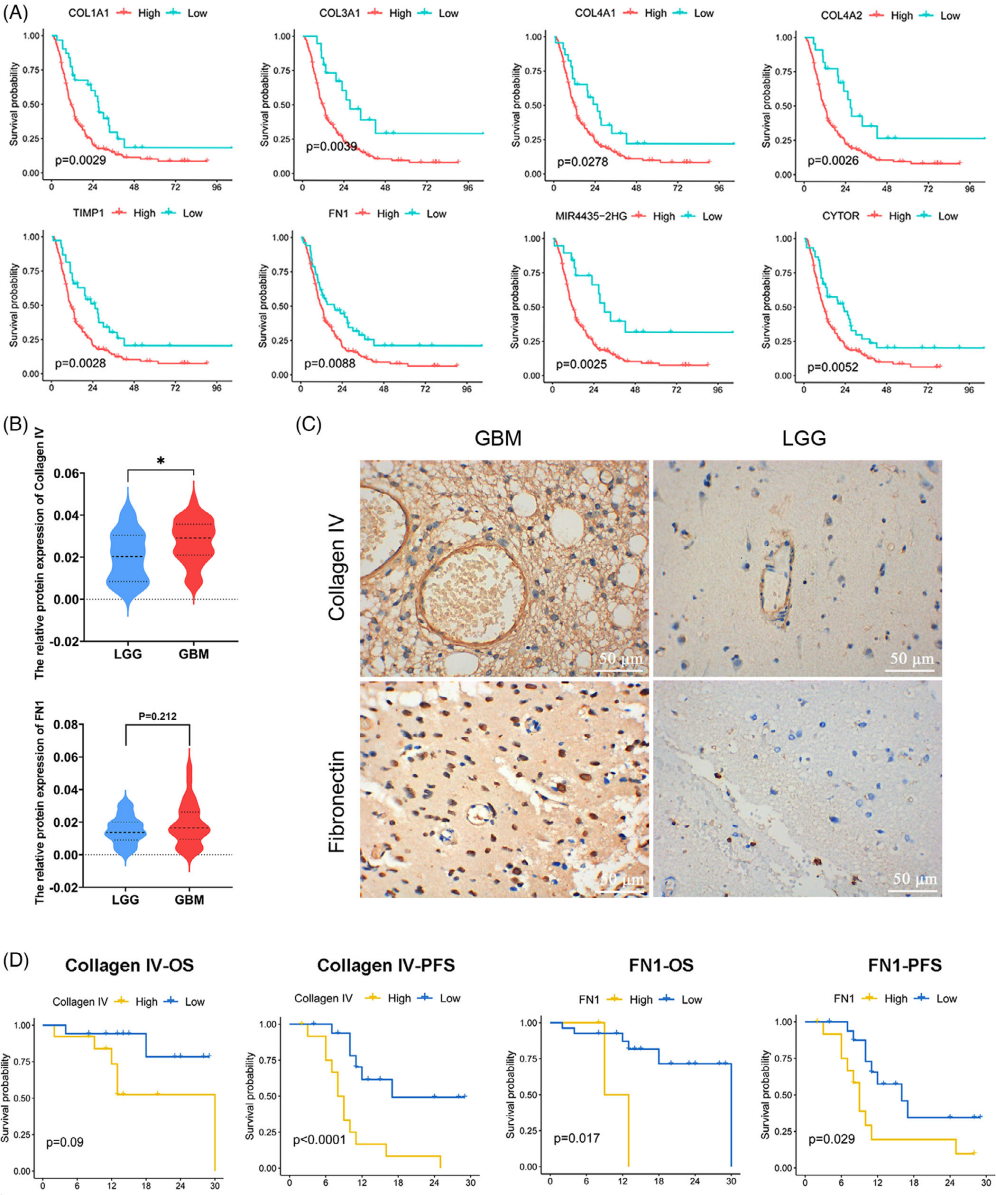
Correlations between DEGs of pericytes in GBM and normal brain tissue and prognosis. (A) Survival curve between the high‐ and low‐expression groups for the top 10 DEGs in the CGGA GBM cohort. (B) Distribution of collagen IV and FN1 expression (quantification of immunohistochemistry [IHC] results) in GBM and low‐grade glioma (LGG) samples (collagen IV: GBM: 0.0274 ± 0.01; LGG: 0.0202 ± 0.012 *p* = 0.026, *n* = 50) (FN1: GBM: 0.018 ± 0.013; LGG: 0.0153 ± 0.008 *p* = 0.212, *n* = 50). (C) IHC results for collagen IV and fibronectin in GBM and LGG samples. (D) Survival curve of the high‐ and low‐gene‐expression groups for collagen IV and FN1, based on IHC results in the GBM cohort.

To explore the interactions between pericytes related to BBTB function and other cells in GBM. The CellPhoneDB analysis showed active interactions between pericytes and other cell types, particularly with other stromal cells (Figure 3A) The pathways exhibiting enrichment in the interaction between PTH1R^+^ pericytes and tumor cells/ECs were of particular interest to us. The FN1–aVb1 and FN1–a5b1 pairs exhibited a high level of enrichment in the interaction between PTH1R^+^ pericytes and ECs, with FN1–aVb1 also showing significant enrichment between PTH1R^+^ pericytes and tumor cells. Collagen–integrin signaling pathways (COL1A2, COL4A1, and COL1A2) were primarily activated between PTH1R^+^ pericytes and other stromal cells (Figure 3B). The interaction pairs in the CGGA GBM cohort identified using CellPhoneDB analysis exhibited significant positive correlations, particularly the extracellular matrix (ECM)–integrin interaction pairs (Figure 3C). Nichenet analysis also revealed active collagen (COL4A1, COL5A3) in PTH1R^+^ pericytes (Figure 3D). Details of the CellPhoneDB and Nichenet analyses are presented in Figure S5. In scRNA‐seq data analysis of paired GBM tumor core and surrounding peripheral tissues, pericytes exhibited localized expression signatures (Figure S6). Analyses of DEGs between pericytes in GBM and nonmalignant tissue also indicated that ECM‐related genes had increased expression levels in tumor tissue (Figure 3E). Notably, the top 10 PTH1R^+^ pericyte DEGs were associated with the prognosis of patients with GBM. *COL1A1*, *COL3A1*, *COL4A1*, *TIMP1*, *COL4A2*, *COL1A1*, *MGP*, *MIR4435‐2HG*, *FN1*, *CYTOR* expression levels were negatively correlated with overall survival (OS), while *THY1* expression levels were positively correlated with OS (Figure 4A). Further stratified analysis showed that these correlations persisted in sex and age subgroups (Figure S7). Interestingly, we found that *COL4A1*, *COL4A2*, and *FN1* not only belonged to the top 10 DEGs between pericytes in GBM and nonmalignant tissue, but these genes in PTH1R^+^ pericytes also showed active interactions with other cells. *COL4A1* and *COL4A2* encode collagen alpha‐1 (IV) and alpha‐2 (IV), which comprise collagen IV, an important component of the BBB basement membrane.^37^ In summary, we primarily explored the relationships between the expression levels of collagen IV/FN1 and the prognosis of patients with GBM. Immunohistochemistry (IHC) staining revealed that the expression level of collagen IV was significantly higher in GBM than low‐grade glioma (LGG), with similar trends observed for FN1 (Figure 4B,C). Within the GBM group, there was a negative correlation between high protein expression of collagen IV and FN1 and both progression‐free survival and OS (Figure 4D). These findings indicate that ECM–integrin signaling pathway proteins (collagen IV and FN1) play important roles in the mechanism of PTH1R^+^ pericytes affecting tumor microenvironment.

### 2.4 The PTH1R expression level correlates with collagen IV and FN1 expression levels and barrier function in the brain

**FIGURE 5:**
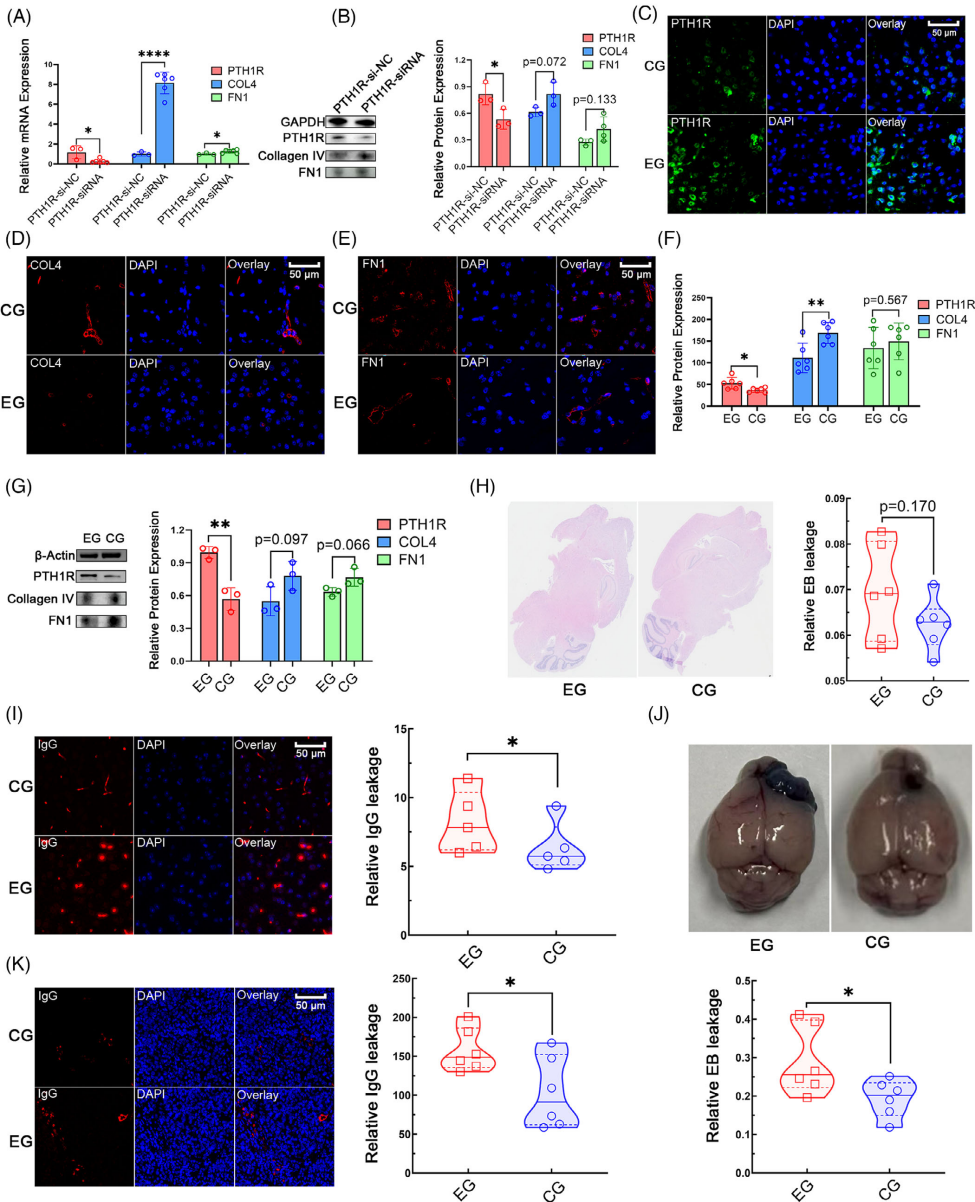
Experimental results: the PTH1R expression level correlates with collagen IV and FN1 expression levels and barrier function in the brain. (A) Quantitative reverse transcription polymerase chain reaction (qRT‐PCR) analysis of the expression levels of *PTH1R*, collagen IV, and *FN1* in the *PTH1R*‐siRNA and NC groups (PTH1R‐siRNA, human brain pericytes transfected with a *PTH1R* small interfering [si]RNA; NC, negative control human brain pericytes; *PTH1R*, difference between means: −0.901 ± 0.274, *p* = 0.0134; *COL4A1*, difference between means: 7.143 ± 0.653, *p* < 0.0001; *FN1*, difference between means: 0.258 ± 0.105, *p* = 0.044, *n* = 3). (B) Western blotting results for PTH1R, collagen IV, and FN1 in the *PTH1R*‐siRNA and NC groups (PTH1R, difference between means: −0.283 ± 0.094, *p* = 0.0134, *n* = 3; COL4A1, difference between mean: 0.202 ± 0.083, *p* = 0.072, *n* = 3; FN1, difference between means: 0.148 ± 0.083, *p* = 0.133, *n* = 3). (C–E) Immunofluorescence (IF) results for PTH1R, collagen IV, and FN1 in mice in the PTH 1–34 administration (EG) and control (CG) groups. (F) IF quantification of PTH1R, collagen IV, and FN1 in EG and CG groups (PTH1R, EG: 52.954 ± 5.409, CG: 37.138 ± 2.082, *p* = 0.021, *n* = 6; collagen IV, EG: 111.319 ± 13.903, CG: 168.846 ± 9.223, *p* = 0.007, *n* = 6; FN1, EG: 133.841 ± 19.456, CG: 149.287 ± 17.342, *p* = 0.567, *n* = 6). (G) Western blotting results for PTH1R, collagen IV, and FN1 in EG and CG groups (PTH1R: difference between means: −0.425 ± 0.068, *p* = 0.003; COL4A1, difference between means: 0.233 ± 0.108, *p* = 0.097, *n* = 3; FN1, difference between means: 0.133 ± 0.053, *p* = 0.066, *n* = 3). (H) Left: the HE dye of brain tissue from the EG and CG groups; right, quantification Evan's Blue (EB) dye leakage and IgG leakage in brain tissue from the EG and CG groups (EB leakage, EG: 0.070 ± 0.004, CG: 0.062 ± 0.006, *p* = 0.170, *n* = 6). (I) Left, IF results of IgG leakage in the EG and CG groups; right, Quantification of IgG leakage in brain tissue from the EG and CG groups (EG: 10.124 ± 1.326, CG: 6.339 ± 0.801, *p* = 0.040, *n* = 5). (J) Up, GL261‐glioma‐bearing mice brain samples from the EG and CG groups; down, quantification Evan's Blue (EB) dye leakage and IgG leakage in brain tissue from the EG and CG groups (EB leakage, EG: 0.290 ± 0.037, CG: 0.194 ± 0.020, *p* = 0.042, *n* = 6). (K) Left, IF results of IgG leakage in the GL261‐glioma‐bearing mice EG and CG groups; right, quantification of IgG leakage in GL261‐glioma‐bearing mice brain tissue from the EG and CG groups (EG: 157.919 ± 27.602, CG: 103.160 ± 46.053, *p* = 0.032, *n* = 6).

To explore whether PTH1R regulates collagen IV and FN1 expression in human brain pericytes (HBPs), we knocked down PTH1R in the HBP cell line HCP. Quantitative reverse transcription‐polymerase chain reaction (qRT‐PCR) analysis showed a significant downregulation of PTH1R expression in the HCP‐PTH1R‐siRNA group compared with the control group, validating the siRNA transfection effect. COL4A1 and FN1 levels were significantly upregulated in the HCP–PTH1R–siRNA cells. The western blotting results confirmed the same trend in PTH1R protein expression levels (Figure 5A,B). PTH1R expression in vivo is known to be promoted by PTH1‐34 administration.^38^, ^39^ After 1 month of PTH 1–34 administration, IF staining revealed increased PTH1R expression levels in mouse brain tissue (Figure 5C). Collagen IV levels were significantly decreased in the PTH 1–34 administration group, and FN1 levels showed the same trend (Figure 5D–F), which was consistent with the results of the cell line experiments. Further western blotting experiments also showed the same trend (Figure 5G). The results of tests using the EB dye in brain tissue indicated that PTH 1–34 administration increased EB leakage in mouse brain tissue compared with that in the control group (Figure 5H). The observed trend suggested weaker barrier function in the PTH 1–34 administration group. IF staining of mouse brain tissue showed increased IgG leakage after PTH 1–34 administration, compared with that in the control group (Figure 5I), which was also indicative of weaker barrier function in the PTH 1–34 administration group. The results in GL261‐glioma‐bearing mice indicated that PTH 1–34 administration increased EB leakage in tumor tissue compared with that in the control group (Figure 5J). Increased IgG leakage in the tumor was also observed in GL261‐glioma‐bearing mice in the PTH 1–34 administration group (Figure 5K). These findings indicate that the PTH1R expression level has negative correlations with collagen IV and FN1 expression levels, and barrier function of the brain.

### 2.5 The predictive value of the tumor pericyte risk score in patients with GBM

**FIGURE 6:**
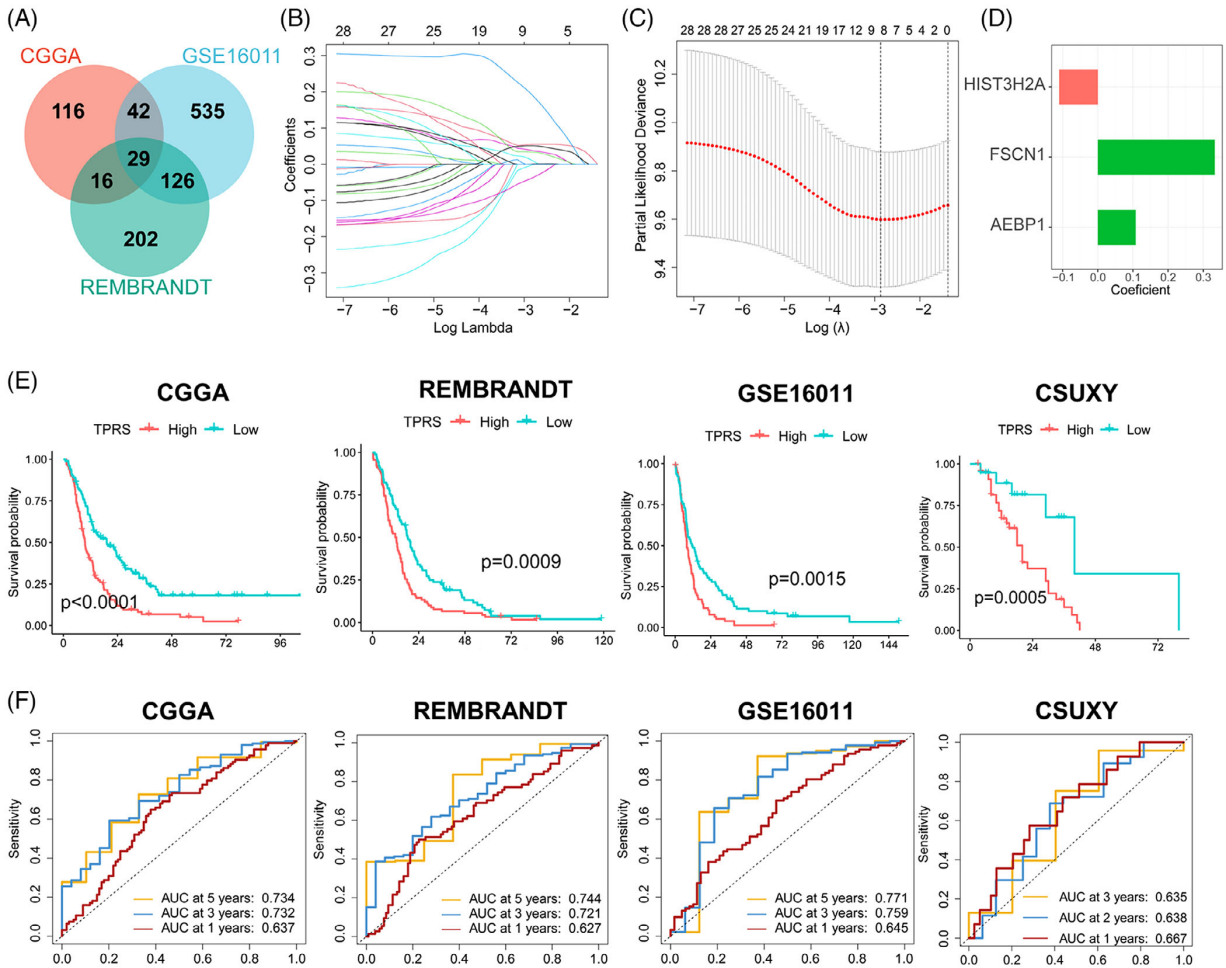
The predictive value of the tumor pericyte risk score on the overall survival of patients with GBM. (A) Intersection of prognostic signature genes in the CGGA, RAMBRANDT, and GSE16011 GBM cohorts. (B) Least absolute shrinkage and selector operation (LASSO) coefficient profiles of 29 prognostic genes. (C) Ten‐fold cross‐validation for tuning parameter selection in the LASSO model for analysis of the correlation between the tumor pericyte risk score (TPRS) and T cell infiltration score in the CGGA GBM cohort. (D) Genes of coefficient in the TPRS. (E) Kaplan–Meier curve of the TPRS‐high and TPRS‐low groups in the GBM cohorts. (F) Time‐dependent receiver operating characteristic (ROC) analysis of the TPRS‐high and TPRS‐low groups in the GBM cohorts.

The prognostic prediction of GBM is important for individualized treatment. The significant prognostic value of tumor pericyte DEGs in GBM was demonstrated in the experiments described above. To further explore the predictive value of pericytes related to BBTB function in GBM, we built a robust prognostic model based on these DEGs to facilitate individualized therapy for GBM. First, we identified tumor pericyte DEGs with significant prognostic value in all three independent cohorts (Figure 6A). Subsequently, we identified eight key prognostic‐related DEGs in the CGGA cohort using least absolute shrinkage and selector operation (LASSO) regression analysis (Figure 6B,C). Finally, using stepwise Cox regression, we created a prognostic model that included three genes (Figure 6D). Using the median value of the tumor pericyte risk score (TPRS) as the risk cut‐off, patients were separated into high‐ and low‐TPRS groups. The low‐TPRS group demonstrated significantly longer OS in four dependent cohorts, including an in‐house GBM cohort (CSUXY) (Figure 6E), and the predictive efficacy of the TPRS was confirmed using receiver operating characteristic curves (Figure 6F). Further stratified analysis of data from the CGGA, GSE16011, and CSUXY cohorts showed that the correlations persisted in different sex and age subgroups (Figure S8). These results indicated that the TPRS can be a useful prognostic model for GBM.

### 2.6 Correlation between the TPRS and immune characteristics in the CGGA cohort

**FIGURE 7:**
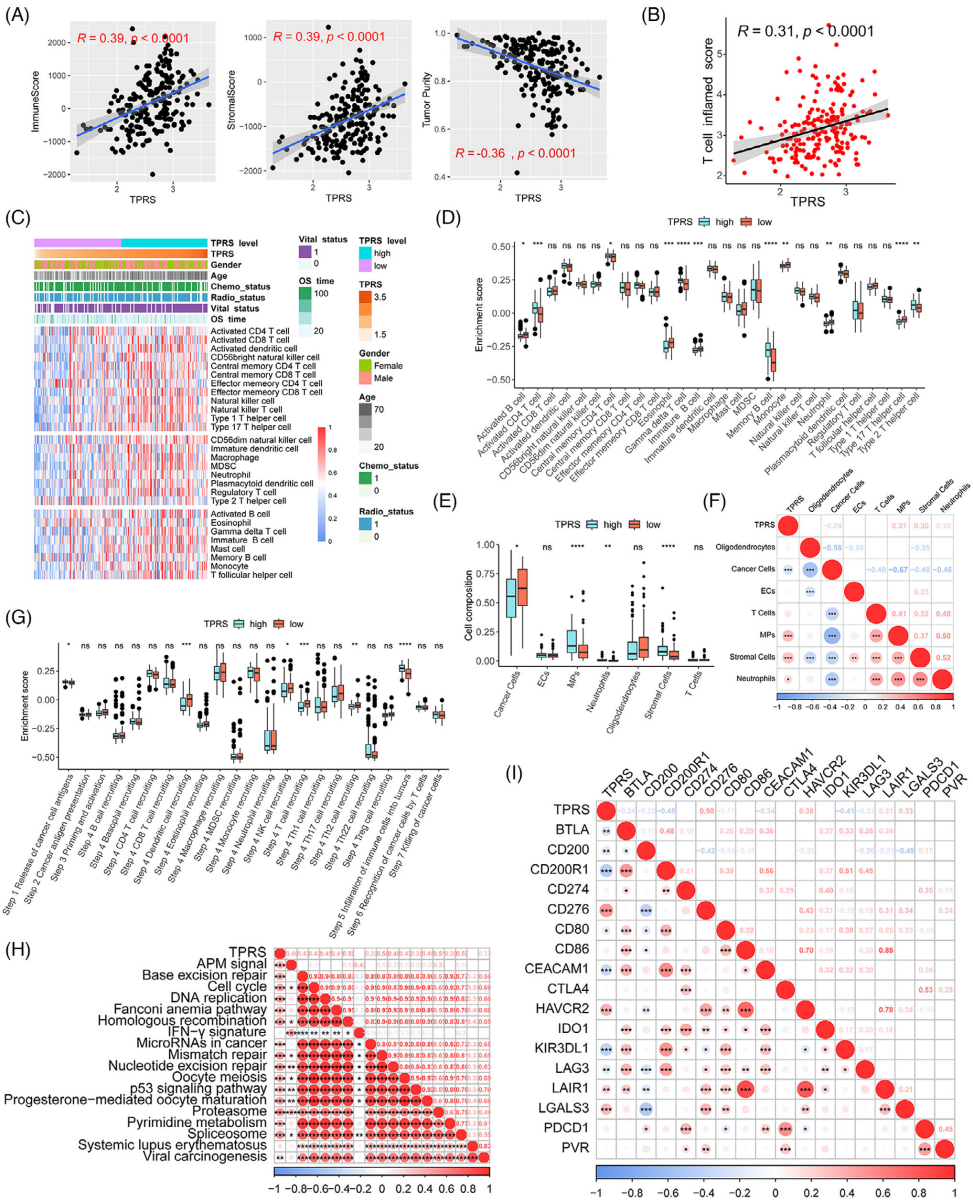
Immune characteristics of the TPRS‐high and TPRS‐low groups. (A) Correlations between the TPRS and the immune score, stromal score, and tumor purity in the CGGA GBM cohort. (B) Correlation between the TPRS and the T cell infiltration score in the CGGA GBM cohort. (C and D) Enrichment of 28 immune cells in the TPRS‐high and TPRS‐low groups in the CGGA cohort. (E and F) CIBERSORTx results of the cell composition and correlation with the TPRS. (G) Enrichment of cancer immunity cycle steps of the TPRS‐high and TPRS‐low groups in the CGGA cohort. (H) Correlations between the TPRS and immunotherapy‐predicted pathways. (I) Correlation between the TPRS with immune checkpoint factors.

Pericytes and other stromal cells play crucial functions in antitumor immunity and potentially impact the choice of immunotherapies, such as mRNA vaccines.^40^ To investigate the correlation between the TPRS and immune characteristics, we analyzed the correlation between the TPRS and the immune score, stromal score, and tumor purity in the CGGA cohort. As illustrated in Figure 7A, we identified significant positive correlations between the TPRS and immune/stromal scores, whereas tumor purity was negatively correlated with the TPRS. Furthermore, as demonstrated in the data presented in Figure 7B, the TPRS was positively correlated with the T cell inflammation score. The enrichment of 28 immune cells was also assessed in the CGGA cohort using the single‐sample GSEA (ssGSEA) method (Figure 7C).^41^ Further analysis revealed significant differences in immune cell components between the TPRS‐high and TPRS‐low groups, with the TPRS‐high group scoring higher for activated CD4^+^ T cells, central memory CD4^+^ T cells, gamma delta T cells, memory B cells, and type 2 T helper cells, while the TPRS‐low group scored higher for activated B cells, eosinophils, immature B cells, monocytes, neutrophils, and type 17 T helper cells (Figure 7D). In addition, we used the CIBERSORTx deconvolution method to estimate differences in the cell composition of tumor tissue between the TPRS‐high and TPRS‐low groups based on scRNA‐seq data (Figure 7E). The abundance of stromal cells, monocytes, and neutrophils was positively correlated with the TPRS, while the abundance of cancer cells was negatively correlated with the TPRS (Figure 7F). The cancer immune cycle objectively demonstrates various immunodulator functions. In the TPRS‐high group, the release of cancer cell antigens (Step 1) and immune cell infiltration into the tumor (Step 5) were more activated. In contrast, dendritic cell recruitment (Step 4), natural killer cell recruitment (Step 4), and Th2 cell recruitment (Step 4) were more activated in the TPRS‐low group (Figure 7G). Moreover, the TPRS was negatively correlated with most immune checkpoint factors, excluding CD276, HAVCR2, LAIR1, and LGALS3. However, the TPRS was positively correlated with most pathways predicted for immunotherapy (Figure 7H–I). These results indicated that the TPRS has close correlation with immune characteristics of GBM.

## 3 DISCUSSION

GBM is a highly malignant brain tumor associated with a poor prognosis. The exclusive nature of the BBB determines the distinct characteristics of this cancer. Drug resistance remains a considerable obstacle to chemotherapy, resulting in a poor prognosis and high recurrence rates. Drug resistance in GBM is exacerbated by the fact that most drug compounds cannot penetrate the BBB and reach the tumor site. Several approaches have been explored over the last few decades to improve the delivery of chemotherapeutic agents to brain tumors, including osmotic BBB disruption, bradykinin‐receptor‐mediated BBB opening, the inhibition of drug efflux transporters, and the exploitation of receptor‐mediated transport systems.^3^ Pericytes are an important component of the BBB, and emerging studies have revealed that pericytes in tumors regulate vascular stability and permeability.^42^ Pericyte coverage is correlated with both tumor invasiveness and immune infiltration, sequentially affecting immune surveillance and promoting tumor metastasis.^43^, ^44^ Pericytes are components of the BBTB and the tumor microenvironment in GBM, contributing to both tumor metastasis and therapeutic resistance.^45^, ^46^, ^47^, ^48^ Utilizing GBM scRNA‐seq data and normal brain pericyte expression signatures, we observed that many other stromal cells, particularly smooth muscle cells, showed the expression of pericyte markers. Although ACTA1, KCNJ8, NG2, PDGFRβ, ABCC1, DLK1, and PROM1 are markers of pericytes, they were expressed in different pericyte clusters (DLK1 and PROM1 in cluster 1; ACTA1 and KCNJ8 in cluster 6; NG2, PDGFRβ, and ABCC1 in cluster 11). We identified a group of pericytes demonstrating significant enrichment in BTBB structure and permeability. PTH1R is a reliable and specific marker for pericytes associated with BTBB functions. Subsequently, the IF costaining of these markers with CD31 proved that their distribution was adjacent to ECs. Pericytes are important targets for overcoming the BTBB.^49^ TGF‐β inhibition reduces the number of αSMA‐positive pericytes, leading to a relaxation in pericyte alignment that allows for enhanced nanoparticle penetration into tumor tissue.^50^ Specific elimination of pericytes in vivo in GBM leads to the collapse of EC walls and vessel lumens, disruption of neovasculature, and inhibited cancer progression.^35^ Targeting pericytes in GBM has the potential to improve prognosis. Further investigation of GBM pericytes and the BBTB will be beneficial for the development of new treatments for GBM.

*PTH1R* encodes the receptor for PTH and PTH‐like hormone. PTH1R is associated with GO terms including the basolateral plasma membrane, basal plasma membrane, and basal part of the cell. While PTH1R has been extensively studied in areas such as skeletal development, angiogenesis, osteoporosis,^51^, ^52^ and Ca2+ homeostasis, its role in BTBB function or the diagnosis and treatment of GBM has not been investigated. This study presents evidence for a potential positive correlation between PTH1R expression levels and BTBB integrity. However, further research is necessary to verify this finding and to determine the specific underlying mechanisms of action. PTH1R^+^ pericytes in GBM tissue demonstrated significant upregulation of ECM‐related genes, and the upregulation of these genes was significantly correlated with GBM grade and a poor prognosis. Collagen IV was identified as the most abundant collagen in the basement membrane. Collagen IV forms a network in the basement membrane, interacting with members of the integrin family, G‐protein‐coupled receptors, and discoidin domain receptors.^53^ This network‐forming collagen is one of the four main component proteins of the basement membrane (collagen IV, laminin, nidogen, and perlecan), which contributes to the integrity of the BBB.^54^ Mutations in COL4A1/2 have been associated with brain malformations and intracerebral hemorrhage.^55^, ^56^, ^57^ This study presents evidence for the association of collagen IV with BBB function. Further research should be undertaken to confirm the observed effect. *FN1* encodes fibronectin, which is a soluble dimeric glycoprotein involved in numerous processes involving cell adhesion and migration, such as embryogenesis, wound healing, blood coagulation, host defense, and metastasis. CellPhoneDB analysis revealed interactions between FN1 and the integrin family as a main pattern of PTH1R^+^ pericyte cell–cell interactions. Furthermore, FN1 was highly expressed in high‐grade gliomas and was its expression level was negatively correlated with GBM prognosis. Integrins are present on every cell type throughout the body, with 24 types of complexes discovered to date.^58^ Integrins bind to their corresponding ECM proteins and play a role in cell proliferation, differentiation, migration, and maintenance.^59^, ^60^ Several ECM–integrin interactions were found to be enriched in PTH1R^+^ pericytes, and these are involved in BBB function. β1‐integrins interact with the ECM proteins laminin, collagen IV, and perlecan and are present in the basal membrane of all microvessels in the brain.^61^ Reducing β1‐integrin expression levels markedly reduced interendothelial claudin‐5 expression levels by confluent or subconfluent ECs in culture. This also led to increased cerebral microvessel permeability in vitro and in vivo, demonstrating the essential role of β1‐integrin function in preserving a fully functioning BBB.^62^ The levels of α5β1 integrin, which has functions related to angiogenesis and tumor development and progression, are significantly upregulated on ECs and astrocytes in multiple tumors.^63^ α5β1 integrin is also linked to resistance to temozolomide in GBM by inhibiting p53 signaling.^64^ It is present on ECs and astrocytes and binds to the ECM components collagen IV and perlecan. The lack of integrin αv in the brain leads to the detachment of astrocytes from the ECM, which in turn increases the permeability of the BBB. Cilengitide, which targets αv/α5 integrins, has recently undergone clinical efficacy testing for the treatment of GBM in combination with temozolomide radiochemotherapy in a phase III clinical study.^65^ These studies indicate significant progress in understanding ECM–integrin signaling in GBM and utilizing this knowledge to develop effective treatments.

In summary, PTH1R^+^ pericytes were associated with BBTB function in GBM. PTH1R^+^ pericytes showed higher expression levels of ECM‐related genes in GBM, which negatively correlated with prognosis. We also designed a TPRS to predict GBM prognosis using the RNA expression profile. However, this study has several limitations. Due to technical limitations, we were unable to separate the pericytes we identified in GBM using a flow cytometer for further experimental validation. Additionally, while the efficiency of the TPRS was validated in other cohorts, it has not yet been validated in prospective cohorts.

## 4 CONCLUSIONS

Using scRNA‐seq data, we identified a type of pericyte highly related to BBTB function in GBM, with PTH1R potentially serving as a marker for these pericytes. PTH1R^+^ pericytes expressed ECM‐related genes, the products of which interact with integrins in other cells within GBM tissue. These pericytes had increased expression levels of ECM‐related genes, such collagen IV and *FN1*. The PTH1R expression level was negatively correlated with collagen IV and FN1 expression levels in the brain and BBB function. The TPRS was found to be a robust prognostic model based on DEGs in PTH1R^+^ pericytes, and its efficiency was validated. This study offers new perspectives and directions for exploring BBTB function and the GBM tumor microenvironment.

## 5 MATERIALS AND METHODS

### 5.1 Patient specimens and publicly available patient cohorts

Nine fresh GBM tissues for scRNA‐seq and paraffin‐embedded tissue specimens of 50 GBMs/LGGs were collected from patients admitted to Xiangya Hospital, Central South University (Figure 1A). Furthermore, 65 GBM samples with complete follow‐up information were collected for RNA‐seq analysis. Written informed consent was obtained from all participants, and the study was approved by the Ethics Committee on Scientific Research of Central South University Xiangya Hospital (approval numbers: 202103150 and 202401003). Publicly available GBM datasets (CGGA, REMBRANDT, and GSE16011^66^) with full clinical information and normalized RNA‐seq data were downloaded from GlioVis (http://gliovis.bioinfo.cnio.es/) and the Gene Expression Omnibus database (https://www.ncbi.nlm.nih.gov/geo/). DEGs of various clusters of normal brain pericytes were obtained from the supplementary data of a previous study.^36^ GBM para‐carcinoma/tumor pairing scRNA‐seq data were obtained from dataset GSE162631.^67^

### 5.2 scRNA‐seq and bulk RNA‐seq analyses

scRNA‐seq was performed using the droplet‐based 10× Genomics platform (10x Genomics, Pleasanton, CA, USA). Rigorous quality control and filtering were performed according to the standard procedure of the “Seurat” package in R studio version 3.6.3, as previously described.^68^ A Seurat file containing transcriptomic data for 93,343 cells derived from nine GBM samples was produced. Total RNA was extracted from tissue samples for bulk RNA‐seq analysis using TRIzol® Reagent according to the manufacturer's instructions. StringTie and RSEM were used for transcript assembly and the quantification of gene expression levels, respectively, and RNA‐seq data were transformed into transcripts per million values. Details of the experimental and analytical methods used for scRNA‐seq and bulk RNA‐seq data analysis are described in the Supporting Information.

### 5.3 Identification of BBTB‐associated pericytes

Pericytes are considered to be stromal cells.^69^ Various pericyte markers were identified through a literature review, and their expression in all stromal cell subclusters were visualized. The 50 most highly upregulated DEGs between brain pericytes and other human vascular cells were identified from supplementary data of a human brain vasculature small nuclear (sn) RNA‐seq study. These were considered as normal brain pericyte expression signatures. GSVA and GSEA were conducted on each stromal cell subcluster using the normal brain pericyte expression signatures. Subclusters with a high enrichment score in GSVA and GSEA and expressing pericyte markers were considered pericytes. GO term functional enrichment was conducted for all subclusters to identify pericytes associated with BBTB function. The 50 most highly upregulated DEGs between BBTB‐related pericytes and other stromal cells were considered as their gene signature. Potential markers for pericytes associated with BBTB function were taken as the intersections between the gene signatures for normal brain pericytes, the gene signatures for pericytes associated with BBTB function, and genes related to the capillary permeability GO term. Pericytes are cells that surrounding the endothelium of blood vessels. GBM samples were costained with CD31 (a recognized marker of ECs) to identify tumor pericytes according to morphological criteria. Multiple IF assays were performed on GBM samples to detect CD31, ANPEP, desmin, and other potential markers to compare the specificity between the potential markers and other pericyte markers (ANPEP and desmin).

### 5.4 Cell culture

Primary HBPs (#1200) were purchased from ScienCell Research Laboratories (Carlsbad, CA, USA) and were cultured in pericyte medium (ScienCellResearch Laboratories).^70^ Experiments were performed between passages 3 and 5.

The GL261 cell line was obtained from Abiowell Biotechnology (https://www.abiowell.com/) and cultured in Dulbecco's modified essential medium (DMEM) supplemented with 10% fetal bovine serum (Gibco, MD, USA).

### 5.5 Parathyroid hormone 1 receptor siRNA transfection

The HBP experimental group was transfected with a small interfering RNA (siRNA; 20 nM; Sangon Biotech, Shanghai, China) against the coding sequence of PTH 1 receptor (*PTH1R*) for 24 h. Transfection of siRNAs was performed overnight at 37°C using Lipofectamine RNAiMax (Life Technologies, Carlsbad, CA, USA), according to the manufacturer's instructions. The siRNA sequence targeting PTH1R was 5′‐CUCCAUUGUGCUCAACU UCTT‐3′ against 5′‐GAAGUUGAGCACAAUGGAGTT‐3′

### 5.6 Quantitative reverse transcription polymerase chain reaction

qRT‐PCR was performed as previously described.^71^ The following primers were used: *PTH1R* forward. 5′‐GCCGGAATGGGACCACATC‐3′ and reverse. 5′‐CGTTC ACGAGTCTCATTGG TG‐3′; *COL4A1* forward, 5′‐GGACTACCTGGAACAAA AGGG‐3′ and reverse 5′‐GCCAAGTATCTCACCTGGATCA‐3′; *FN1* forward, 5′‐CGGTGGCTGTCAGTCAA AG‐3′ and reverse, 5′‐AAACCTCGGCTTCCTCCATAA‐3′. qRT‐PCR was performed using a LightCycler 480 apparatus (Roche Diagnostics, Basel, Switzerland) using the LightCycler 480 SYBR Green IMaster kit (Roche Diagnostics).

### 5.7 Animals and experimental design

Male C57BLN/6 mice (6–8 weeks old, weighing 23–28 g; RRID: IMSR JAX:000664) were obtained from the Charles River Laboratories (Beijing, China). The animals were housed in an individually ventilated cage system under controlled temperature and humidity conditions, with a 12‐h/12‐h light/dark cycle at 25°C, and had free access to food and tap water. The study adhered to the ARRIVE (Animal Research: Reporting In Vivo Experiments) guidelines and complied with the Guidelines for the Care and Use of Laboratory Animals in Biomedical Research.^72^

GL261‐glioma‐bearing models were established using these mice. The mice were kept under deep anesthesia with 2% isoflurane during surgery. Using a stereotactic apparatus, a single‐cell suspension of GL261 cells (80,000 cells in 1 µL of DMEM) was implanted into the right striatum at a rate of 0.25 µL per minute.

Sample size calculations were not performed. A total of 20 naive mice were arbitrarily assigned to one of two groups: a parathyroid hormone (PTH 1–34) administration group and a control group. PTH 1–34 administration increased the PTH1R expression level in vivo; therefore, it was administered to the experimental group.^38^, ^39^ Normal saline (control group) or PTH 1–34 (100 µg/kg/d) was subcutaneously administered to the mice (*n* = 20, 10 animals per group) every day for 1 month as previously described.^73^ Twelve GL261‐glioma‐bearing mice were arbitrarily assigned to the PTH 1–34 administration group or the control group (*n* = 6 per group). Normal saline or PTH 1–34 (100 µg/kg/d) was subcutaneously administered to GL261‐glioma‐bearing mice every day for 3 weeks due to the short life expectancy of GL261‐glioma‐bearing mice.

### 5.8 BBB function assessment

To detect BBB function in the PTH 1–34 administration and control groups, Evans Blue (EB #E2129)^74^ and IgG leakage detection^75^ were performed. For EB leakage detection, mice were injected with 4 mL/kg of 2% EB solution in 0.9% saline via the caudal vein. After 10 min, the mice were deeply anesthetized with isoflurane and perfused with ice‐cold PBS to clear the dye from the blood vessels. The mice were then sacrificed by rapid cervical dislocation under deep anesthesia, and the brain tissue was quickly harvested after decapitation. Brain tissue was weighed and cut into 3–5 µm‐thick sections. The sections were then immersed in formamide solution (#W6104191000) at 50°C for 72 h to extract the EB. After the homogenates were centrifuged at 5000×*g* for 5 min at 4°C, the resulting supernatant was diluted fourfold with a solution containing ethanol, and the fluorescence intensity was measured using a microplate fluorescence reader (TECAN, Männedorf, Switzerland). High EB leakage indicated weak BBB function. IgG leakage detection was based on the quantification of IF staining of the brain tissue sections. High IgG leakage also indicated weak BBB function.

### 5.9 Immunohistochemistry

IHC was performed as previously described.^76^ The tissue sections were incubated with 0.3% hydrogen peroxide for 10 min at room temperature and washed twice with PBS. The tissue sections were then blocked with 5% goat serum for 10 min, washed, and incubated overnight with the primary antibody at 4°C. They were then incubated with secondary antibody (1:400; Abcam, Cambridge, MA, USA) at 37°C for 30 min. The sections were then developed using a DAB substrate kit (Sangon Biotech) and counterstained with hematoxylin (Sangon Biotech). The immunohistochemical score was calculated as the integrated optical density/area. The following primary antibodies were used: rabbit polyclonal anticollagen IV (Abcam; ab6586, 1:100 dilution) and rabbit polyclonal antifibronectin (Abcam; ab2413, 1:100 dilution) antibodies.

### 5.10 Immunofluorescence staining

Free‐floating sections were first blocked with a buffer containing 10% goat serum, 1% bovine serum albumin, and 0.3% Triton X‐100 in PBS for 1 h, followed by overnight incubation with selected antibodies in blocking buffer at 4°C. The following day, the sections were washed three times with PBS and incubated with the appropriate fluorescent secondary antibodies (Jackson ImmunoResearch, West Baltimore, USA) diluted 1:250 in blocking buffer for 1 h at 25°C. The slides were visualized using a fluorescence microscope. The following primary antibodies were used: rabbit monoclonal antibody [EPR25414‐249] against ATP1A3 + alpha 1 sodium potassium ATPase + ATP1A2 + ATP1A4 (Abcam; ab300507, 1:100 dilution); anti‐GATA1(Ab‐142) antibody (Signalway Antibody; 21041–2, 1:100 dilution); anti‐PTHR1 antibody (Affinity; DF6589, 1:100 dilution); mouse monoclonal [JC/70A] anti‐CD31 antibody (Abcam; ab9498, 1:200 dilution); rabbit polyclonal anticollagen‐IV antibody (Abcam; ab6586, 1:100 dilution); rabbit polyclonal antifibronectin antibody (Abcam; ab2413, 1:100 dilution); rabbit polyclonal anti‐CD13 antibody (Servicebio; GB11356‐100, 1:100 dilution); and recombinant antidesmin antibody (Servicebio; GB15075‐100, 1:100 dilution). The results were documented using an FV500 upright confocal microscope (AXlO; ZEISS, Oberkochen, Germany).

### 5.11 Development of a TPRS

First, we analyzed the prognostic value of pericyte‐associated DEGs in three independent cohorts using univariate Cox regression analysis. Relevant DEGs with prognostic value in all three cohorts were screened using Venn diagrams. Subsequently, risk models were constructed using data from the CGGA cohort, as previously documented.^77^ Briefly, the prognosis‐relevant DEGs were downscaled for further screening using the LASSO algorithm. The final model was then constructed, and coefficients determined using stepwise Cox regression. The modeling equation was as follows:

TPRS=Σβi∗RNAi,

where *βi* is the coefficient of the gene in the risk model, and 𝑅𝑁𝐴𝑖 is the gene expression value. Specifically, patients were classified into high‐ and low‐TPRS groups based on the median TPRS. The Kaplan–Meier method was applied, and the log‐rank test was used to compare the groups to estimate the prognostic significance of the TPRS. The statistical performance of the TPRS was assessed using the tROC R package. Next, two independent cohorts (REMBRANDT and GSE16011) and an in‐house cohort (CSUXY) were used as external validation sets to validate the performance of the TPRS.

### 5.12 Immunological characterization

The ESTIMATE algorithm^78^ was used, as previously described,^79^ to calculate the overall immune and stromal cell abundance and quantify tumor purity for each sample. A set of pan‐cancer T‐cell‐inflammation‐associated signatures was collected from Ayers et al.,^80^ to define pre‐existing cancer immunity and predict the clinical response to immune checkpoint inhibitors (ICIs). In addition, a gene set for the cancer immune cycle, including seven steps,^81^ was collected. A gene set of 28 immune cells was obtained from a previous study by Charoentong et al.^41^ A set of gene profiles positively associated with the clinical response to ICIs was collected from a previous study by Mariathasan.^82^ The enrichment scores of the above gene sets were quantified using ssGSEA using the R package “GSVA,” as previously described.^83^

### 5.13 Statistical analysis

All data analyses were performed using R software, version 3.6.3. Correlations between variables were measured using Pearson's or Spearman's coefficients. The Student's *t*‐test was used for continuous variables with a normal distribution, while the Mann–Whitney *U* test was used for variables with a non‐normal distribution. Categorical variables were compared using the chi‐square test or Fisher's exact test. Survival curves for prognostic analyses were generated using the Kaplan–Meier method, and the log‐rank test was applied to estimate statistical significance. The significance level was set at *p* < 0.05, and all statistical tests were two‐sided.

## AUTHOR CONTRIBUTIONS

*Conception and design*: Yuzhe Li, Changwu Wu, and Qing Liu. *Acquisition and analysis of data (provided tissue microarrays, statistical analysis, and biostatistics)*: Yuzhe Li, Changwu Wu, and Minghua Wu. *In vitro and in vivo experiments*: Yuzhe Li and Changwu Wu. *Writing, review, and/or revision of the manuscript*: Yuzhe Li, Changwu Wu, Xinmiao Long, Xiangyu Wang, Wei Gao, Kun Deng, Bo Xie, and Sen Zhang. *Study supervision*: Qing Liu and Minghua Wu. All authors read and approved the final version of the manuscript.

## ETHICS STATEMENT

This research was approved by the Ethics Committee on Scientific Research of Central South University Xiangya Hospital (for clinical experiments approval numbers: 202103150; for animal experiments approval numbers: 202401003). Written informed consent was obtained from all participants.

## CONFLICT OF INTEREST STATEMENT

The authors declare that they have no conflict of interest.

## Supporting information



Supporting Information

## Data Availability

Normalized RNA sequencing (RNA‐seq) data from the CGGA, REMBRANDT, GSE162631, and GSE16011 datasets were downloaded from GlioVis (http://gliovis.bioinfo.cnio.es/) and the Gene Expression Omnibus database (https://www.ncbi.nlm.nih.gov/geo/). The glioma cohort, CSUXY, has been uploaded to the China National Center for Bioinformation database (https://ngdc.cncb.ac.cn/gsa, GSA: HRA007149). The single‐cell (sc) RNA‐seq data were analyzed by Singleron Biotechnologies CO., Ltd. (Nanjing, China). The raw scRNA‐seq data reported in this paper have also been deposited in the Genome Sequence Archive in the National Genomics Data Center, China National Center for Bioinformation, Chinese Academy of Sciences (GSA‐Human: HRA007138), publicly accessible at https://ngdc.cncb.ac.cn/gsa‐human. Other information is available from the corresponding author upon reasonable request.

## References

[1] Alexander BM , Cloughesy TF . Adult glioblastoma. J Clin Oncol. 2017;35(21):2402‐2409. 28640706 10.1200/JCO.2017.73.0119

[2] Wu W , Klockow JL , Zhang M , et al. Glioblastoma multiforme (GBM): an overview of current therapies and mechanisms of resistance. Pharmacol Res. 2021;171:105780. 34302977 10.1016/j.phrs.2021.105780 PMC8384724

[3] van Tellingen O , Yetkin‐Arik B , de Gooijer MC , Wesseling P , Wurdinger T , de Vries HE . Overcoming the blood‐brain tumor barrier for effective glioblastoma treatment. Drug Resist Updat. 2015;19:1‐12. 25791797 10.1016/j.drup.2015.02.002

[4] Langen UH , Ayloo S , Gu C . Development and cell biology of the blood‐brain barrier. Annu Rev Cell Dev Biol. 2019;35:591‐613. 31299172 10.1146/annurev-cellbio-100617-062608 PMC8934576

[5] Rong L , Li N , Zhang Z . Emerging therapies for glioblastoma: current state and future directions. J Exp Clin Cancer Res. 2022;41(1):142. 35428347 10.1186/s13046-022-02349-7 PMC9013078

[6] Liu Y , Wang W , Zhang D , et al. Brain co‐delivery of first‐line chemotherapy drug and epigenetic bromodomain inhibitor for multidimensional enhanced synergistic glioblastoma therapy. Exploration (Beijing). 2022;2(4):20210274. 37325609 10.1002/EXP.20210274 PMC10190947

[7] Matsuo M , Miwa K , Tanaka O , et al. Impact of [11C]methionine positron emission tomography for target definition of glioblastoma multiforme in radiation therapy planning. Int J Radiat Oncol Biol Phys. 2012;82(1):83‐89. 21095072 10.1016/j.ijrobp.2010.09.020

[8] Pafundi DH , Laack NN , Youland RS , et al. Biopsy validation of 18F‐DOPA PET and biodistribution in gliomas for neurosurgical planning and radiotherapy target delineation: results of a prospective pilot study. Neuro Oncol. 2013;15(8):1058‐1067. 23460322 10.1093/neuonc/not002 PMC3714146

[9] Toyokawa G , Seto T , Takenoyama M , Ichinose Y . Insights into brain metastasis in patients with ALK+ lung cancer: is the brain truly a sanctuary?. Cancer Metastasis Rev. 2015;34(4):797‐805. 26342831 10.1007/s10555-015-9592-y PMC4661196

[10] Hardee ME , Zagzag D . Mechanisms of glioma‐associated neovascularization. Am J Pathol. 2012;181(4):1126‐1141. 22858156 10.1016/j.ajpath.2012.06.030 PMC3463636

[11] Vásquez X , Sánchez‐Gómez P , Palma V . Netrin‐1 in glioblastoma neovascularization: the new partner in crime?. Int J Mol Sci. 2021;22(15):8248. 34361013 10.3390/ijms22158248 PMC8348949

[12] Tavora B , Mederer T , Wessel KJ , et al. Tumoural activation of TLR3‐SLIT2 axis in endothelium drives metastasis. Nature. 2020;586(7828):299‐304. 32999457 10.1038/s41586-020-2774-y PMC8088828

[13] Paiva AE , Lousado L , Almeida VM , et al. Endothelial cells as precursors for osteoblasts in the metastatic prostate cancer bone. Neoplasia. 2017;19(11):928‐931. 28957694 10.1016/j.neo.2017.08.007 PMC5619995

[14] Prazeres P , Turquetti AOM , Azevedo PO , et al. Perivascular cell αv integrins as a target to treat skeletal muscle fibrosis. Int J Biochem Cell Biol. 2018;99:109‐113. 29627438 10.1016/j.biocel.2018.04.002 PMC6159891

[15] Caporarello N , D'Angeli F , Cambria MT , et al. Pericytes in microvessels: from “mural” function to brain and retina regeneration. Int J Mol Sci. 2019;20(24):6351. 31861092 10.3390/ijms20246351 PMC6940987

[16] Longden TA , Zhao G , Hariharan A , Lederer WJ . Pericytes and the control of blood flow in brain and heart. Annu Rev Physiol. 2023;85:137‐164. 36763972 10.1146/annurev-physiol-031522-034807 PMC10280497

[17] Giannoni P , Badaut J , Dargazanli C , et al. The pericyte‐glia interface at the blood‐brain barrier. Clin Sci (Lond). 2018;132(3):361‐374. 29439117 10.1042/CS20171634

[18] Attwell D , Mishra A , Hall CN , O'Farrell FM , Dalkara T . What is a pericyte?. J Cereb Blood Flow Metab. 2016;36(2):451‐455. 26661200 10.1177/0271678X15610340 PMC4759679

[19] Guerra DAP , Paiva AE , Sena IFG , et al. Targeting glioblastoma‐derived pericytes improves chemotherapeutic outcome. Angiogenesis. 2018;21(4):667‐675. 29761249 10.1007/s10456-018-9621-x PMC6238207

[20] Hart DA . One of the primary functions of tissue‐resident pluripotent pericytes cells may be to regulate normal organ growth and maturation: implications for attempts to repair tissues later in life. Int J Mol Sci. 2022;23(10):5496. 35628309 10.3390/ijms23105496 PMC9146368

[21] Andreotti JP , Paiva AE , Prazeres P , et al. The role of natural killer cells in the uterine microenvironment during pregnancy. Cell Mol Immunol. 2018;15(11):941‐943. 29572543 10.1038/s41423-018-0023-1 PMC6207766

[22] Shavit‐Stein E , Berkowitz S , Gofrit SG , Altman K , Weinberg N , Maggio N . Neurocoagulation from a mechanistic point of view in the central nervous system. Semin Thromb Hemost. 2022;48(3):277‐287. 35052009 10.1055/s-0041-1741569

[23] Rustenhoven J , Aalderink M , Scotter EL , et al. TGF‐beta1 regulates human brain pericyte inflammatory processes involved in neurovasculature function. J Neuroinflammation. 2016;13:37. 26867675 10.1186/s12974-016-0503-0 PMC4751726

[24] Zhu S , Chen M , Ying Y , et al. Versatile subtypes of pericytes and their roles in spinal cord injury repair, bone development and repair. Bone Res. 2022;10(1):30. 35296645 10.1038/s41413-022-00203-2 PMC8927336

[25] Santos GSP , Magno LAV , Romano‐Silva MA , Mintz A , Birbrair A . Pericyte plasticity in the brain. Neurosci Bull. 2019;35(3):551‐560. 30367336 10.1007/s12264-018-0296-5 PMC6527663

[26] Asada N , Kunisaki Y , Pierce H , et al. Differential cytokine contributions of perivascular haematopoietic stem cell niches. Nat Cell Biol. 2017;19(3):214‐223. 28218906 10.1038/ncb3475 PMC5467892

[27] Nishiyama A , Boshans L , Goncalves CM , Wegrzyn J , Patel KD . Lineage, fate, and fate potential of NG2‐glia. Brain Res. 2016;1638:116‐128. Pt B. 26301825 10.1016/j.brainres.2015.08.013 PMC4761528

[28] Wohl SG , Schmeer CW , Friese T , Witte OW , Isenmann S . In situ dividing and phagocytosing retinal microglia express nestin, vimentin, and NG2 in vivo. PLoS One. 2011;6(8):e22408. 21850226 10.1371/journal.pone.0022408 PMC3151247

[29] Cao Z , Liu Y , Wang Y , Leng P . Research progress on the role of PDGF/PDGFR in type 2 diabetes. Biomed Pharmacother. 2023;164:114983. 37290188 10.1016/j.biopha.2023.114983

[30] Öhlund D , Handly‐Santana A , Biffi G , et al. Distinct populations of inflammatory fibroblasts and myofibroblasts in pancreatic cancer. J Exp Med. 2017;214(3):579‐596. 28232471 10.1084/jem.20162024 PMC5339682

[31] Klinkhammer BM , Floege J , Boor P . PDGF in organ fibrosis. Mol Aspects Med. 2018;62:44‐62. 29155002 10.1016/j.mam.2017.11.008

[32] Morikawa S , Baluk P , Kaidoh T , Haskell A , Jain RK , McDonald DM . Abnormalities in pericytes on blood vessels and endothelial sprouts in tumors. Am J Pathol. 2002;160(3):985‐1000. 11891196 10.1016/S0002-9440(10)64920-6 PMC1867175

[33] Yamazaki T , Young KH . Effects of radiation on tumor vasculature. Mol Carcinog. 2022;61(2):165‐172. 34644811 10.1002/mc.23360

[34] Ellison‐Hughes GM , Madeddu P . Exploring pericyte and cardiac stem cell secretome unveils new tactics for drug discovery. Pharmacol Ther. 2017;171:1‐12. 27916652 10.1016/j.pharmthera.2016.11.007 PMC5636619

[35] Cheng L , Huang Z , Zhou W , et al. Glioblastoma stem cells generate vascular pericytes to support vessel function and tumor growth. Cell. 2013;153(1):139‐152. 23540695 10.1016/j.cell.2013.02.021 PMC3638263

[36] Garcia FJ , Sun N , Lee H , et al. Single‐cell dissection of the human brain vasculature. Nature. 2022;603(7903):893‐899. 35158371 10.1038/s41586-022-04521-7 PMC9680899

[37] Kumar AA , Yeo N , Whittaker M , et al. Vascular collagen type‐IV in hypertension and cerebral small vessel disease. Stroke. 2022;53(12):3696‐3705. 36205142 10.1161/STROKEAHA.122.037761 PMC9698121

[38] Tirado‐Cabrera I , Martin‐Guerrero E , Heredero‐Jimenez S , Ardura JA , Gortázar AR . PTH1R translocation to primary cilia in mechanically‐stimulated ostecytes prevents osteoclast formation via regulation of CXCL5 and IL‐6 secretion. J Cell Physiol. 2022;237(10):3927‐3943. 35933642 10.1002/jcp.30849 PMC9804361

[39] Znorko B , Pawlak D , Oksztulska‐Kolanek E , et al. RANKL/OPG system regulation by endogenous PTH and PTH1R/ATF4 axis in bone: implications for bone accrual and strength in growing rats with mild uremia. Cytokine. 2018;106:19‐28. 29529595 10.1016/j.cyto.2018.03.002

[40] Xie L , Wang G , Sang W , et al. Phenolic immunogenic cell death nanoinducer for sensitizing tumor to PD‐1 checkpoint blockade immunotherapy. Biomaterials. 2021;269:120638. 33421711 10.1016/j.biomaterials.2020.120638

[41] Charoentong P , Finotello F , Angelova M , et al. Pan‐cancer immunogenomic analyses reveal genotype‐immunophenotype relationships and predictors of response to checkpoint blockade. Cell Rep. 2017;18(1):248‐262. 28052254 10.1016/j.celrep.2016.12.019

[42] Östman A , Corvigno S . Microvascular mural cells in cancer. Trends Cancer. 2018;4(12):838‐848. 30470305 10.1016/j.trecan.2018.10.004

[43] Hong J , Tobin NP , Rundqvist H , et al. Role of tumor pericytes in the recruitment of myeloid‐derived suppressor cells. J Natl Cancer Inst. 2015;107(10):djv209. 26296362 10.1093/jnci/djv209 PMC6592827

[44] Tian L , Goldstein A , Wang H , et al. Mutual regulation of tumour vessel normalization and immunostimulatory reprogramming. Nature. 2017;544(7649):250‐254. 28371798 10.1038/nature21724 PMC5788037

[45] Kong D , Kwon D , Moon B , et al. CD19 CAR‐expressing iPSC‐derived NK cells effectively enhance migration and cytotoxicity into glioblastoma by targeting to the pericytes in tumor microenvironment. Biomed Pharmacother. 2024;174:116436. 38508081 10.1016/j.biopha.2024.116436

[46] Park S , Avera AD , Kim Y . Biomanufacturing of glioblastoma organoids exhibiting hierarchical and spatially organized tumor microenvironment via transdifferentiation. Biotechnol Bioeng. 2022;119(11):3252‐3274. 35869574 10.1002/bit.28191

[47] Bonde AK , Tischler V , Kumar S , Soltermann A , Schwendener RA . Intratumoral macrophages contribute to epithelial‐mesenchymal transition in solid tumors. BMC Cancer. 2012;12:35. 22273460 10.1186/1471-2407-12-35 PMC3314544

[48] Barcellos‐Hoff MH , Lyden D , Wang TC . The evolution of the cancer niche during multistage carcinogenesis. Nat Rev Cancer. 2013;13(7):511‐518. 23760023 10.1038/nrc3536

[49] Fujimoto T , Nakagawa S , Morofuji Y , et al. Pericytes suppress brain metastasis from lung cancer in vitro. Cell Mol Neurobiol. 2020;40(1):113‐121. 31414300 10.1007/s10571-019-00725-0 PMC11448953

[50] Kano MR , Bae Y , Iwata C , et al. Improvement of cancer‐targeting therapy, using nanocarriers for intractable solid tumors by inhibition of TGF‐beta signaling. Proc Natl Acad Sci USA. 2007;104(9):3460‐3465. 17307870 10.1073/pnas.0611660104 PMC1800736

[51] Santa Maria C , Cheng Z , Li A , et al. Interplay between CaSR and PTH1R signaling in skeletal development and osteoanabolism. Semin Cell Dev Biol. 2016;49:11‐23. 26688334 10.1016/j.semcdb.2015.12.004 PMC4761456

[52] Zhai X , Mao C , Shen Q , et al. Molecular insights into the distinct signaling duration for the peptide‐induced PTH1R activation. Nat Commun. 2022;13(1):6276. 36271004 10.1038/s41467-022-34009-x PMC9586930

[53] Chew C , Lennon R . Basement membrane defects in genetic kidney diseases. Front Pediatr. 2018;6:11. 29435440 10.3389/fped.2018.00011 PMC5796894

[54] Patino MG , Neiders ME , Andreana S , Noble B , Cohen RE . Collagen: an overview. Implant Dent. 2002;11(3):280‐285. 12271567 10.1097/00008505-200207000-00014

[55] Favor J , Gloeckner CJ , Janik D , et al. Type IV procollagen missense mutations associated with defects of the eye, vascular stability, the brain, kidney function and embryonic or postnatal viability in the mouse, Mus musculus: an extension of the Col4a1 allelic series and the identification of the first two Col4a2 mutant alleles. Genetics. 2007;175(2):725‐736. 17179069 10.1534/genetics.106.064733 PMC1800636

[56] Kuo DS , Labelle‐Dumais C , Mao M , et al. Allelic heterogeneity contributes to variability in ocular dysgenesis, myopathy and brain malformations caused by Col4a1 and Col4a2 mutations. Hum Mol Genet. 2014;23(7):1709‐1722. 24203695 10.1093/hmg/ddt560 PMC3943517

[57] Jeanne M , Labelle‐Dumais C , Jorgensen J , et al. COL4A2 mutations impair COL4A1 and COL4A2 secretion and cause hemorrhagic stroke. Am J Hum Genet. 2012;90(1):91‐101. 22209247 10.1016/j.ajhg.2011.11.022 PMC3257894

[58] Takada Y , Ye X , Simon S . The integrins. Genome Biol. 2007;8(5):215. 17543136 10.1186/gb-2007-8-5-215 PMC1929136

[59] Hynes RO . Integrins: versatility, modulation, and signaling in cell adhesion. Cell. 1992;69(1):11‐25. 1555235 10.1016/0092-8674(92)90115-s

[60] Iwamoto DV , Calderwood DA . Regulation of integrin‐mediated adhesions. Curr Opin Cell Biol. 2015;36:41‐47. 26189062 10.1016/j.ceb.2015.06.009 PMC4639423

[61] Yamada KM , Miyamoto S . Integrin transmembrane signaling and cytoskeletal control. Curr Opin Cell Biol. 1995;7(5):681‐689. 8573343 10.1016/0955-0674(95)80110-3

[62] Osada T , Gu YH , Kanazawa M , et al. Interendothelial claudin‐5 expression depends on cerebral endothelial cell‐matrix adhesion by β(1)‐integrins. J Cereb Blood Flow Metab. 2011;31(10):1972‐1985. 21772312 10.1038/jcbfm.2011.99 PMC3208159

[63] Schaffner F , Ray AM , Dontenwill M . Integrin α5β1, the fibronectin receptor, as a pertinent therapeutic target in solid tumors. Cancers (Basel). 2013;5(1):27‐47. 24216697 10.3390/cancers5010027 PMC3730317

[64] Janouskova H , Maglott A , Leger DY , et al. Integrin α5β1 plays a critical role in resistance to temozolomide by interfering with the p53 pathway in high‐grade glioma. Cancer Res. 2012;72(14):3463‐3470. 22593187 10.1158/0008-5472.CAN-11-4199

[65] Stupp R , Hegi ME , Gorlia T , et al. Cilengitide combined with standard treatment for patients with newly diagnosed glioblastoma with methylated MGMT promoter (CENTRIC EORTC 26071‐22072 study): a multicentre, randomised, open‐label, phase 3 trial. Lancet Oncol. 2014;15(10):1100‐1108. 25163906 10.1016/S1470-2045(14)70379-1

[66] Gravendeel LA , Kouwenhoven MC , Gevaert O , et al. Intrinsic gene expression profiles of gliomas are a better predictor of survival than histology. Cancer Res. 2009;69(23):9065‐9072. 19920198 10.1158/0008-5472.CAN-09-2307

[67] Xie Y , He L , Lugano R , et al. Key molecular alterations in endothelial cells in human glioblastoma uncovered through single‐cell RNA sequencing. JCI Insight. 2021;6(15):e150861. 34228647 10.1172/jci.insight.150861 PMC8410070

[68] Chen Z , Zhou L , Liu L , et al. Single‐cell RNA sequencing highlights the role of inflammatory cancer‐associated fibroblasts in bladder urothelial carcinoma. Nat Commun. 2020;11(1):5077. 33033240 10.1038/s41467-020-18916-5 PMC7545162

[69] Avraham S , Korin B , Chung JJ , Oxburgh L , Shaw AS . The Mesangial cell—the glomerular stromal cell. Nat Rev Nephrol. 2021;17(12):855‐864. 34508249 10.1038/s41581-021-00474-8

[70] Navarro R , Delgado‐Wicke P , Nuñez‐Prado N , et al. Role of nucleotide‐binding oligomerization domain 1 (NOD1) in pericyte‐mediated vascular inflammation. J Cell Mol Med. 2016;20(5):980‐986. 26915562 10.1111/jcmm.12804 PMC4831361

[71] Wu C , Tan J , Shen H , et al. Exploring the relationship between metabolism and immune microenvironment in osteosarcoma based on metabolic pathways. J Biomed Sci. 2024;31(1):4. 38212768 10.1186/s12929-024-00999-7 PMC10785352

[72] Jones‐Bolin S . Guidelines for the care and use of laboratory animals in biomedical research. Curr Protoc Pharmacol. 2012. Appendix 4:Appendix 4B. 10.1002/0471141755.pha04bs59 23258596

[73] Fu X , Zhou B , Yan Q , et al. Kindlin‐2 regulates skeletal homeostasis by modulating PTH1R in mice. Signal Transduct Target Ther. 2020;5(1):297. 33361757 10.1038/s41392-020-00328-y PMC7762753

[74] Lindenau KL , Barr JL , Higgins CR , Sporici KT , Brailoiu E , Brailoiu GC . Blood‐brain barrier disruption mediated by FFA1 Receptor‐evidence using miniscope. Int J Mol Sci. 2022;23(4):2258. 35216375 10.3390/ijms23042258 PMC8875452

[75] Li S , Kumar TP , Joshee S , et al. Endothelial cell‐derived GABA signaling modulates neuronal migration and postnatal behavior. Cell Res. 2018;28(2):221‐248. 29086765 10.1038/cr.2017.135 PMC5799810

[76] Wu C , Su J , Wang X , et al. Overexpression of the phospholipase A2 group V gene in glioma tumors is associated with poor patient prognosis. Cancer Manag Res. 2019;11:3139‐3152. 31114356 10.2147/CMAR.S199207 PMC6489671

[77] Wu C , Long W , Qin C , et al. Liquid biopsy‐based identification of prognostic and immunotherapeutically relevant gene signatures in lower grade glioma. J Big Data. 2023;10(1):19.

[78] Yoshihara K , Shahmoradgoli M , Martínez E , et al. Inferring tumour purity and stromal and immune cell admixture from expression data. Nat Commun. 2013;4:2612. 24113773 10.1038/ncomms3612 PMC3826632

[79] Wu C , Tan J , Wang X , et al. Pan‐cancer analyses reveal molecular and clinical characteristics of cuproptosis regulators. iMeta. 2023;2(1):e68. 38868340 10.1002/imt2.68 PMC10989956

[80] Ayers M , Lunceford J , Nebozhyn M , et al. IFN‐γ‐related mRNA profile predicts clinical response to PD‐1 blockade. J Clin Invest. 2017;127(8):2930‐2940. 28650338 10.1172/JCI91190 PMC5531419

[81] Chen DS , Mellman I . Oncology meets immunology: the cancer‐immunity cycle. Immunity. 2013;39(1):1‐10. 23890059 10.1016/j.immuni.2013.07.012

[82] Mariathasan S , Turley SJ , Nickles D , et al. TGFβ attenuates tumour response to PD‐L1 blockade by contributing to exclusion of T cells. Nature. 2018;554(7693):544‐548. 29443960 10.1038/nature25501 PMC6028240

[83] Wu C , Qin C , Long W , Wang X , Xiao K , Liu Q . Tumor antigens and immune subtypes of glioblastoma: the fundamentals of mRNA vaccine and individualized immunotherapy development. J Big Data. 2022;9(1):92. 35855914 10.1186/s40537-022-00643-x PMC9281265

[84] Huang FJ , You WK , Bonaldo P , Seyfried TN , Pasquale EB , Stallcup WB . Pericyte deficiencies lead to aberrant tumor vascularizaton in the brain of the NG2 null mouse. Dev Biol. 2010;344(2):1035‐1046. 20599895 10.1016/j.ydbio.2010.06.023 PMC3197744

[85] Bondjers C , He L , Takemoto M , et al. Microarray analysis of blood microvessels from PDGF‐B and PDGF‐Rbeta mutant mice identifies novel markers for brain pericytes. Faseb J. 2006;20(10):1703‐1705. 16807374 10.1096/fj.05-4944fje

[86] Oishi K , Kamiyashiki T , Ito Y . Isometric contraction of microvascular pericytes from mouse brain parenchyma. Microvasc Res. 2007;73(1):20‐28. 17030042 10.1016/j.mvr.2006.08.004

[87] Cho H , Kozasa T , Bondjers C , Betsholtz C , Kehrl JH . Pericyte‐specific expression of Rgs5: implications for PDGF and EDG receptor signaling during vascular maturation. Faseb J. 2003;17(3):440‐442. 12514120 10.1096/fj.02-0340fje

[88] Yang AC , Stevens MY , Chen MB , et al. Physiological blood‐brain transport is impaired with age by a shift in transcytosis. Nature. 2020;583(7816):425‐430. 32612231 10.1038/s41586-020-2453-z PMC8331074

[89] Crisan M , Corselli M , Chen WC , Péault B . Perivascular cells for regenerative medicine. J Cell Mol Med. 2012;16(12):2851‐2860. 22882758 10.1111/j.1582-4934.2012.01617.x PMC4393715

[90] Guerra‐Rebollo M , Garrido C , Sánchez‐Cid L , et al. Targeting of replicating CD133 and OCT4/SOX2 expressing glioma stem cells selects a cell population that reinitiates tumors upon release of therapeutic pressure. Sci Rep. 2019;9(1):9549. 31267022 10.1038/s41598-019-46014-0 PMC6606606

[91] Göritz C , Dias DO , Tomilin N , Barbacid M , Shupliakov O , Frisén J . A pericyte origin of spinal cord scar tissue. Science. 2011;333(6039):238‐242. 21737741 10.1126/science.1203165

[92] Kunisaki Y , Bruns I , Scheiermann C , et al. Arteriolar niches maintain haematopoietic stem cell quiescence. Nature. 2013;502(7473):637‐643. 24107994 10.1038/nature12612 PMC3821873

[93] Guerra DAP , Paiva AE , Sena IFG , et al. Adipocytes role in the bone marrow niche. Cytometry A. 2018;93(2):167‐171. 29236351 10.1002/cyto.a.23301 PMC6067923

[94] Sena IFG , Borges IT , Lousado L , et al. LepR+ cells dispute hegemony with Gli1+ cells in bone marrow fibrosis. Cell Cycle. 2017;16(21):2018‐2022. 28976809 10.1080/15384101.2017.1367072 PMC5731410

[95] Christian S , Winkler R , Helfrich I , et al. Endosialin (Tem1) is a marker of tumor‐associated myofibroblasts and tumor vessel‐associated mural cells. Am J Pathol. 2008;172(2):486‐494. 18187565 10.2353/ajpath.2008.070623 PMC2312374

[96] Kunz J , Krause D , Kremer M , Dermietzel R . The 140‐kDa protein of blood‐brain barrier‐associated pericytes is identical to aminopeptidase N. J Neurochem. 1994;62(6):2375‐2386. 7910634 10.1046/j.1471-4159.1994.62062375.x

[97] Huang Q , Liu L , Xiao D , et al. CD44(+) lung cancer stem cell‐derived pericyte‐like cells cause brain metastases through GPR124‐enhanced trans‐endothelial migration. Cancer Cell. 2023;41(9):1621‐1636. e1628. 37595587 10.1016/j.ccell.2023.07.012

[98] Danopoulos S , Bhattacharya S , Mariani TJ , Al Alam D . Transcriptional characterisation of human lung cells identifies novel mesenchymal lineage markers. Eur Respir J. 2020;55(1):1900746. 31619469 10.1183/13993003.00746-2019 PMC8055172

[99] Wakui S , Yokoo K , Muto T , et al. Localization of Ang‐1, ‐2, Tie‐2, and VEGF expression at endothelial‐pericyte interdigitation in rat angiogenesis. Lab Invest. 2006;86(11):1172‐1184. 16969369 10.1038/labinvest.3700476

[100] Cheng Q , Tang A , Wang Z , et al. CALD1 modulates gliomas progression via facilitating tumor angiogenesis. Cancers (Basel). 2021;13(11):2705. 34070840 10.3390/cancers13112705 PMC8199308

[101] Zhou W , Chen C , Shi Y , et al. Targeting glioma stem cell‐derived pericytes disrupts the blood‐tumor barrier and improves chemotherapeutic efficacy. Cell Stem Cell. 2017;21(5):591‐603. e594. 29100012 10.1016/j.stem.2017.10.002 PMC5687837

