# Supplementary material for: Single‐cell transcriptomic analysis of glioblastoma reveals pericytes contributing to the blood–brain–tumor barrier and tumor progression
Source: MedComm (2020). 2024 Dec 4;5(12):e70014. doi: 10.1002/mco2.70014 (PMC11617595; doi:10.1002/mco2.70014)
Supplement: Supplementary file 1 — Supporting Information [file MCO2-5-e70014-s001.pdf]

Single-Cell Transcriptomic Analysis of Glioblastoma Reveals Pericytes Contributing to the Blood–Brain–Tumor Barrier and Tumor Progression

Running title: Blood–Brain–Tumor Barrier and GBM Pericytes

Yuzhe Li[1,3#], Changwu Wu[1,4#], Xinmiao Long[2], Xiangyu Wang[1], Wei Gao[2], Kun Deng[2], Bo Xie[1], Sen Zhang[1], Minghua Wu[1,2*], Qing Liu[1,4*]

#Yuzhe Li and Changwu Wu contributed equally to this work.

[1]Department of Neurosurgery, Xiangya Hospital, Central South University, Changsha, Hunan 410008, China.

[2]Cancer Research Institute of Central South University, Changsha, Hunan 410008, China.

[3]Chinese Academy of Medical Sciences and Peking Union Medical College, Beijing 100037, China.

[4]National Clinical Research Center for Geriatric Disorders, Xiangya Hospital, Central South University, Changsha, Hunan, China.

***Corresponding Author**

Dr. Liu Qing

Email: liuqingdr@csu.edu.cn

Dr. Minghua Wu

Email: wuminghua554@aliyun.com

Supplementary methods

Single-cell suspension preparation

The fresh GBM tissues were stored in sCelLiveTM Tissue Preservation Solution (Singleron) on ice within 30 mins after resection. The tissues were washed three times with Hanks Balanced Salt Solution (HBSS), minced and then digested with 3 mL sCelLiveTM Tissue Dissociation Solution (Singleron) in Singleron PythoN™ Tissue Dissociation System at 37 °C for 15 min. Cell suspension was filtered through a 40-micron sterile strainer, then incubated with GEXSCOPE® red blood cell lysis buffer (RCLB, Singleron)[Cell: RCLB=1:2 (volume ratio)] at room temperature for 5-8 min. The mixture was then centrifuged for 5 mins at 300 × g 4 ℃ to remove the supernatant.

Droplet-based scRNA-seq

A concentration of 1×10^5 cells ml^{-1} were converted to barcoded scRNA-seq libraries according to the protocols of the Chromium Single Cell 5'Library, Gel Bead & Multiplex Kit (10x Genomics). The cells were partitioned into Gel Beads in Emulsion in the ChromiumTM Controller instrument where cell lysis and barcoded reverse transcription of RNA occurred. All the libraries were sequenced on Illumina Novaseq 6000 with 150 bp paired end reads.

RT & Amplification & Library Construction

The Singleron Matrix® Single Cell Processing System was used to load single cell

suspensions (2×10^5 cells/mL) in PBS (HyClone) onto a microwell chip. Barcoding beads were then collected from the microwell chip and used to capture mRNA, which was reverse transcribed to produce cDNA. PCR amplification was performed on the amplified cDNA, which was then fragmented and ligated with sequencing adapters. The scRNA-seq libraries were constructed using the GEXSCOPE® Single Cell RNA Library Kits (Singleron) protocol [1], and individual libraries were diluted to 4 nM, pooled, and sequenced on an Illumina NovaSeq 6000 platform using 150 bp paired-end reads.

Primary analysis of raw read data

For primary analysis of the raw read data, the CeleScope (v1.9.0) pipeline was used to generate gene expression matrices. Low quality reads were removed using Cutadapt v1.17[2] to trim poly-A tail and adapter sequences. Cell barcode and unique molecular identifier (UMI) information was extracted, and STAR v2.6.1a [3] was used to map the reads to the GRCh38 reference genome (ensembl version 92 annotation). UMI and gene counts for each cell were obtained using featureCounts v2.0.1[4] software and used to generate expression matrix files for downstream analysis.

Quality control, dimension reduction and clustering

We applied strict filtering criteria to exclude cells with low gene counts (<200), high mitochondrial content (>50%), and low UMI counts (bottom 2%). After filtering, a total of 121,637 high quality cells were retained for subsequent analysis, with an average of

2372 genes and 6328 UMIs per cell. To reduce the dimensionality of the data, we used functions from Seurat v3.1.2 [5] to perform PCA analysis of the top 2000 variable genes identified using the FindVariableFeatures() function. We then used the first 20 principal components to identify cell clusters using the FindClusters() function. The Harmony algorithm [6] was used to remove batch effects between samples.

Statistics and reproducibility

To assess the statistical significance of our results, we performed unpaired two-tailed Wilcoxon rank-sum tests to compare cell distribution between two groups, unpaired two-tailed Student's t-tests to compare gene expression or gene signature between two groups of cells, and paired two-tailed Wilcoxon rank-sum tests to compare cell distribution of paired celltype1 and celltype2. All statistical analyses were performed using R, and the significance level was set at $p < 0.05$.

Differentially expressed genes analysis

Analysis of differentially expressed genes (DEGs) was performed using the Seurat FindMarkers function with default parameters and a Wilcox likelihood ratio test. We selected genes as DEGs that were expressed in more than 10% of the cells in a cluster and had an average log(fold change) value greater than 0.25. Cell type annotation of each cluster was performed by combining the expression of canonical markers found in the DEGs with literature knowledge. Heatmaps, dot plots, and violin plots were generated using Seurat DoHeatmap, DotPlot, and Vlnplot functions, respectively, to

display the expression of markers for each cell type. Doublet cells were identified as those expressing markers for different cell types and were manually removed.

Cell type annotation

The cell type identity of each cluster was determined using canonical markers found in the DEGs and the SynEcoSys database. Heatmaps, dot plots and violin plots were generated using Seurat v3.1.2 DoHeatmap, DotPlot and Vlnplot functions to display the expression of markers used to identify each cell type.

Pathway enrichment analysis

To uncover the possible functions of the differentially expressed genes (DEGs), we performed pathway enrichment analysis using the R package "clusterProfiler" 3.16.1 [7] with databases such as Gene Ontology (GO) and Kyoto Encyclopedia of Genes and Genomes (KEGG). We considered pathways with a p-adjusted value of less than 0.05 to be significantly enriched. As a reference, we used GO gene sets including molecular function (MF), biological process (BP), and cellular component (CC) categories. To further investigate the DEGs between two groups, we performed Gene Set Enrichment Analysis (GSEA) and Gene Set Variation Analysis (GSVA) pathway enrichment analysis using the GSVA package [8]. For GSVA analysis, we used the average gene expression of each cell type as input data.

Trajectory analysis

We used Monocle2 and RNA velocity to achieve trajectory analysis. In Moncle2 analysis cell were sorted based on differentially expressed genes to reconstruct the trajectory of cell differentiation, resulting in a spatio-temporal order of differentiation [9]. We used DDRTree for dimension reduction and FindVariableFeatures, and visualized the trajectory using the plot_cell_trajectory() function. Additionally, we used CytoTRACE to predict the differentiation potential of monocyte subpopulations based on gene counts and expression from single-cell RNA-sequencing data [10].

For RNA velocity analysis, we used velocyto v0.2.3 and scVelo v0.17.17 in Python with default parameters [11], using a BAM file containing stromal cells and the reference genome GRCh38 (hg38).

Cell-cell interaction analysis

CellPhoneDB v2.1.0[12] was used and focused on known receptor-ligand interactions between two cell types or subtypes. To determine the null distribution of the average ligand-receptor expression levels of the interacting clusters, the cluster labels of all cells were randomly permuted 1000 times. Individual ligand or receptor expression was evaluated using a cutoff based on the average log gene expression distribution for all genes across all cell types. Significant cell-cell interactions were identified by a p-value < 0.05 and an average log expression > 0.1. These interactions were visualized using the R package circlize v0.4.10.

NicheNet (v1.1.1) was used to rank the ligands based on how well they predicted whether a gene was linked with a gene set of interest compared to the background gene

set. DEGs between pericytes associated to BBTB functions and other stromal cells were defined as the gene set of interest. All other genes expressed in the receiver tissue (average mean expression over all conditions > 1 tag/kb) were considered background.

Single cell-based deconvolution

To evaluate the cell composition in TPRS high and low groups, CIBERSORTx (https://cibersortx.stanford.edu/) analysis was performed using cell subpopulations identified based on scRNA-Seq in this study. The correlation between TPRS and cell composition was also conducted.

Bulk RNA-Seq of GBM samples

Tissue samples were extracted for total RNA using TRIzol® Reagent according to the manufacturer's instructions. RNA quality was evaluated using the 5300 Bioanalyzer, and quantification was performed with the ND-2000. Library preparation and sequencing were performed by Shanghai Majorbio Bio-pharm Biotechnology Co, Ltd. using the Illumina® Stranded mRNA Prep, Ligation kit. The RNA-seq library was prepared with 1μg of total RNA through polyA selection, fragmentation, and cDNA synthesis using random hexamer primers. The resulting cDNA underwent end-repair following Illumina's library construction protocol. After PCR amplification and quantification, paired-end RNA-seq library was sequenced using the NovaSeq 6000 sequencer. Raw reads were trimmed and quality-controlled using fastp, and alignment to the reference genome was performed with HISAT2. StringTie and RSEM were used

for read assembly and quantification of gene expression levels, respectively. Finally, the RNA-Seq data was transformed into TPM.

References

1. Dura, B., et al., *scFTD-seq: freeze-thaw lysis based, portable approach toward highly distributed single-cell 3' mRNA profiling.* Nucleic Acids Res, 2019. **47**(3): p. e16.
2. Kechin, A., et al., *cutPrimers: A New Tool for Accurate Cutting of Primers from Reads of Targeted Next Generation Sequencing.* J Comput Biol, 2017. **24**(11): p. 1138-1143.
3. Dobin, A., et al., *STAR: ultrafast universal RNA-seq aligner.* Bioinformatics, 2013. **29**(1): p. 15-21.
4. !!! INVALID CITATION !!! .
5. Satija, R., et al., *Spatial reconstruction of single-cell gene expression data.* Nat Biotechnol, 2015. **33**(5): p. 495-502.
6. Korsunsky, I., et al., *Fast, sensitive and accurate integration of single-cell data with Harmony.* Nat Methods, 2019. **16**(12): p. 1289-1296.
7. Yu, G., et al., *clusterProfiler: an R package for comparing biological themes among gene clusters.* Omics, 2012. **16**(5): p. 284-7.
8. Hänzelmann, S., R. Castelo, and J. Guinney, *GSVA: gene set variation analysis for microarray and RNA-seq data.* BMC Bioinformatics, 2013. **14**: p. 7.
9. Qiu, X., et al., *Single-cell mRNA quantification and differential analysis with Census.* Nat Methods, 2017. **14**(3): p. 309-315.
10. Gulati, G.S., et al., *Single-cell transcriptional diversity is a hallmark of developmental potential.* Science, 2020. **367**(6476): p. 405-411.
11. Bergen, V., et al., *Generalizing RNA velocity to transient cell states through dynamical modeling.* Nat Biotechnol, 2020. **38**(12): p. 1408-1414.
12. Efremova, M., et al., *CellPhoneDB: inferring cell-cell communication from combined expression of multi-subunit ligand-receptor complexes.* Nat Protoc, 2020. **15**(4): p. 1484-1506.

Supplementary Figures.

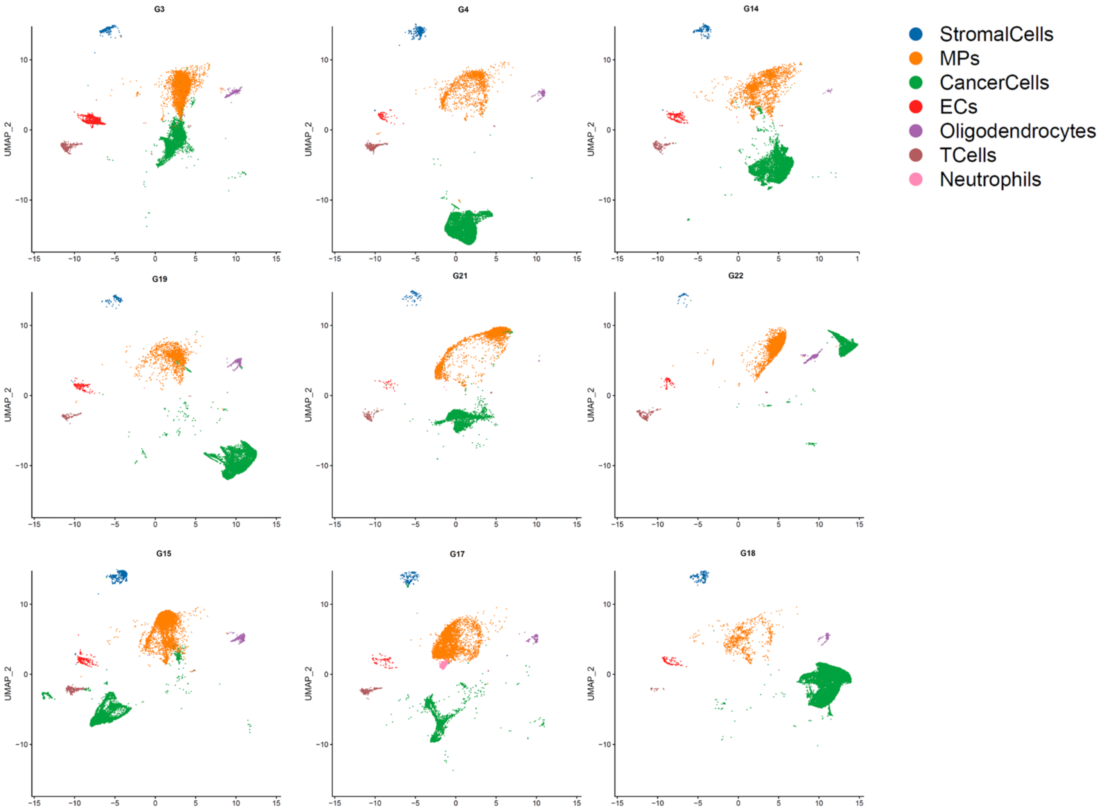

Figure S1 Cellular component of each sample visualized in UMAP plot.

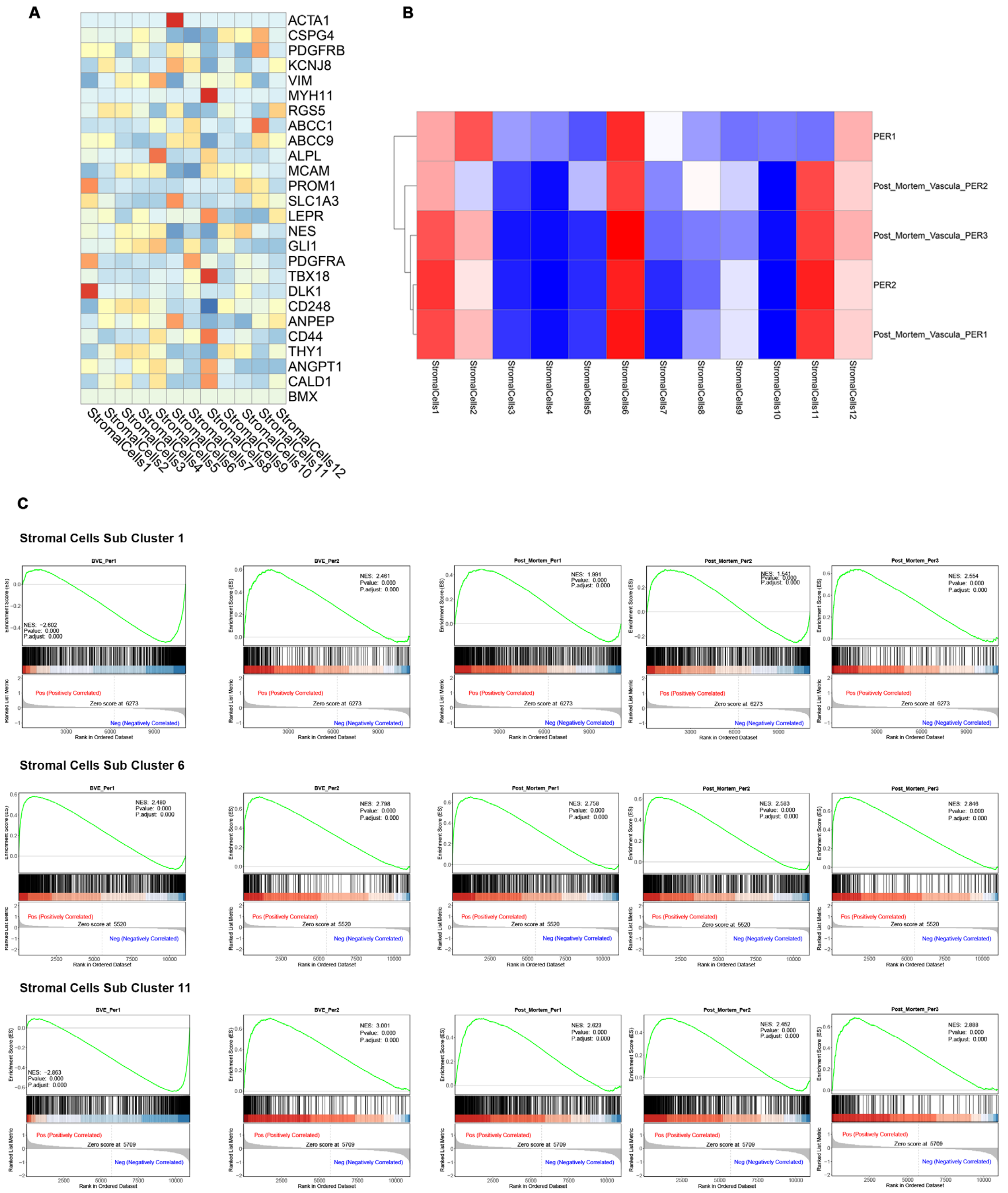

Figure S2 Verification of the pericyte annotation in the GBM. **A** Expression of pericytes markers from a previous study in stromal cell sub clusters. **B** GSVA of stromal cell sub clusters compared with normal brain pericytes gene expression signatures. (PER 1: top 50 DEGs of aSMCs-like pericytes comparing with other vascular cells in brain; PER 2: top 50 DEGs of vSMCs-like pericytes compared with other vascular cells in brain; Post_Mortem_Vascula_PER1, Post_Mortem_Vascula_PER2, and

Post_Mortem_ Vascula-PER3 are three pericytes identified from postmortem tissue data, top 50 DEGs of three postmortem pericytes with other vascular cells were used as well, these five expression profiles are defined as normal brain pericytes gene expression signatures). **C** GSEA performed with normal brain pericyte gene expression signatures with stromal cell sub cluster 1, 6, 8, and 11, showing significant results in GSVA.

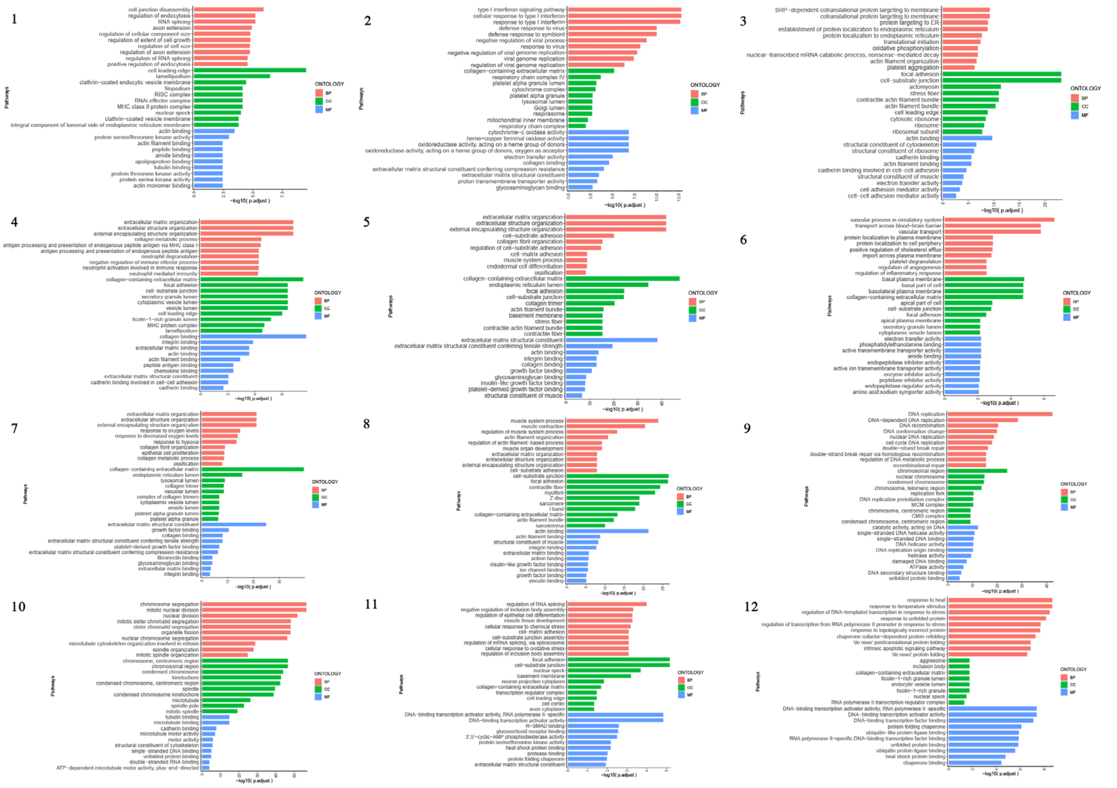

Figure S3 GO enrichment analysis of stromal cell sub clusters DEGs presented in the bar chart. DEGs used in were GO enrichment analysis from each stromal cell sub cluster comparing with other stromal cells.

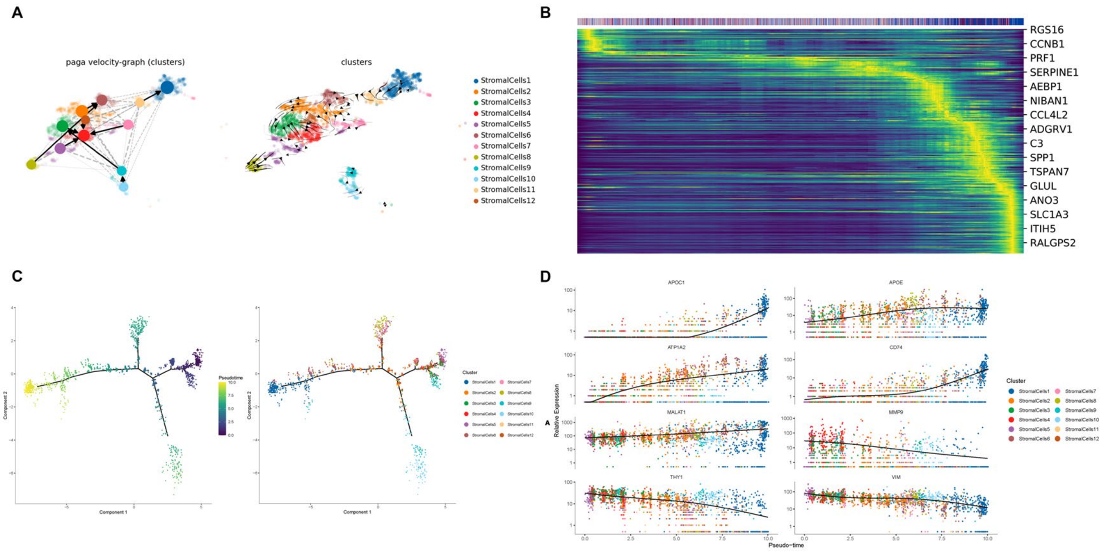

Figure S4 Trajectory analysis of stromal cell sub clusters. **A** PAGA-velocity graph of stromal cells, with arrows representing the direction of cell flow. **B** Top genes of RNA velocity analysis in the heatmap. **C** Trajectory analysis of stromal cell sub clusters in Monocle2. **D** Differentially expressed genes used in Monocle2.

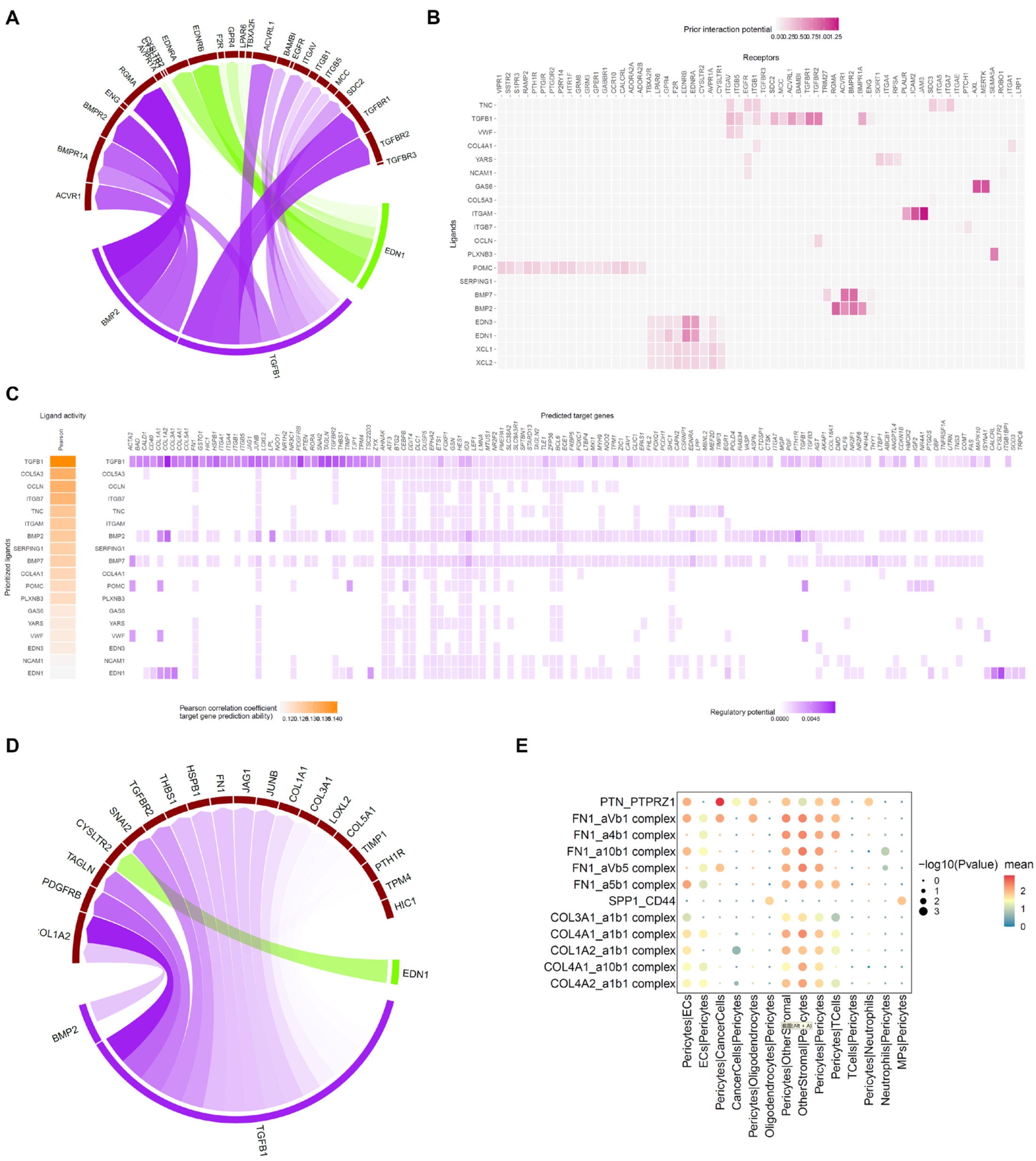

Figure S5 Cell-cell interaction analysis. **A** Circos plot of the active receptor ligand in the Nichenet analysis. **B** Heatmap of receptor ligand activity in the Nichenet analysis. **C** Heatmap of target ligand activity in the Nichenet analysis. **D** Circos plot of the active target ligand in the Nichenet analysis. **E** Significant interaction pair between PTH1R+ pericyte and other cells in CellPhoneDB.

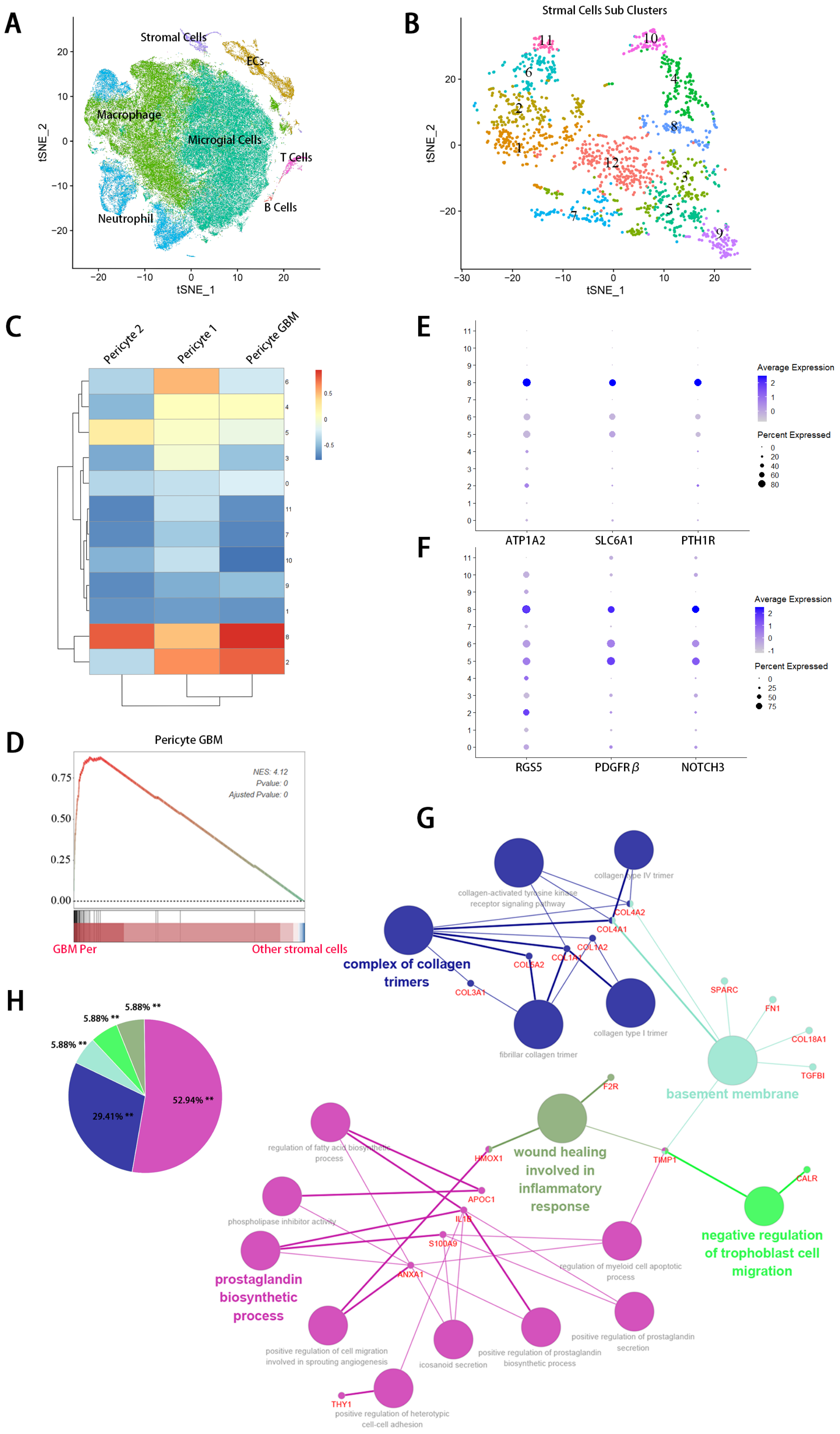
A
Stromal Cells
ECs
Macrophage
Microglial Cells
T Cells
B Cells
Neutrophil
tSNE_1
tSNE_2
B
Strmal Cells Sub Clusters
tSNE_1
tSNE_2
C
Pericyte 2
Pericyte 1
Pericyte GBM
D
Pericyte GBM
NES: 4.12
Pvalue: 0
Ajusted Pvalue: 0
GBM Per
Other stromal cells
E
ATP1A2
SLC6A1
PTH1R
Average Expression
Percent Expressed
F
RGS5
PDGFR β
NOTCH3
Average Expression
Percent Expressed
G
complex of collagen trimers
collagen-activated tyrosine kinase receptor signaling pathway
collagen type IV trimer
collagen type I trimer
fibrillar collagen trimer
basement membrane
wound healing involved in inflammatory response
negative regulation of trophoblast cell migration
prostaglandin biosynthetic process
regulation of fatty acid biosynthetic process
phospholipase inhibitor activity
regulation of myeloid cell apoptotic process
positive regulation of prostaglandin secretion
positive regulation of cell migration involved in sprouting angiogenesis
icosanoid secretion
positive regulation of prostaglandin biosynthetic process
positive regulation of heterotypic cell-cell adhesion
COL4A2
COL4A1
COL1A2
COL1A1
COL5A2
COL3A1
SPARC
FN1
COL18A1
TGFBI
F2R
TIMP1
CALR
HMOX1
APOC1
IL1B
S100A9
ANXA1
THY1
H
52.94% **
29.41% **
5.88% **
5.88% **
5.88% **

223

Figure S6 Difference between pericytes in GBM and normal brain tissue. **A** ScRNA-seq data of 4 GBM tumor/ tumor-adjacent tissue pair visualized in the t-SNE plot. **B** Sub clusters of stromal cells in the t-SNE plot. **C** GSVA of stromal cell sub clusters comparing pericyte expression signatures (Pericyte GBM: top 50 DEGs of PTH1R$^+$pericytes compared with other stromal cells in GBM; Pericyte 1: top 50 DEGs of aSMCs-like pericytes compared with other vascular cells in brain; Pericyte 2: top 50 DEGs of vSMCs-like pericytes compared with other vascular cells in brain). **D** GSEA of stromal cell cluster 8 in PTH1R$^+$ pericytes expression signatures. **E** Expression of potential markers (ATP1A2, SLC6A1, and PTH1R) in sub clusters of stromal cells. **F** expression of previous recognized hallmarks (RGS5, PDGFRβ, and NOTCH3) in sub clusters of stromal cells. **G** GO enrichment analysis of DEGs between pericytes in GBM and normal brain tissue presented in network chart. **H** GO enrichment analysis of DEGs between pericytes in GBM and normal brain tissue presented in the pie chat.

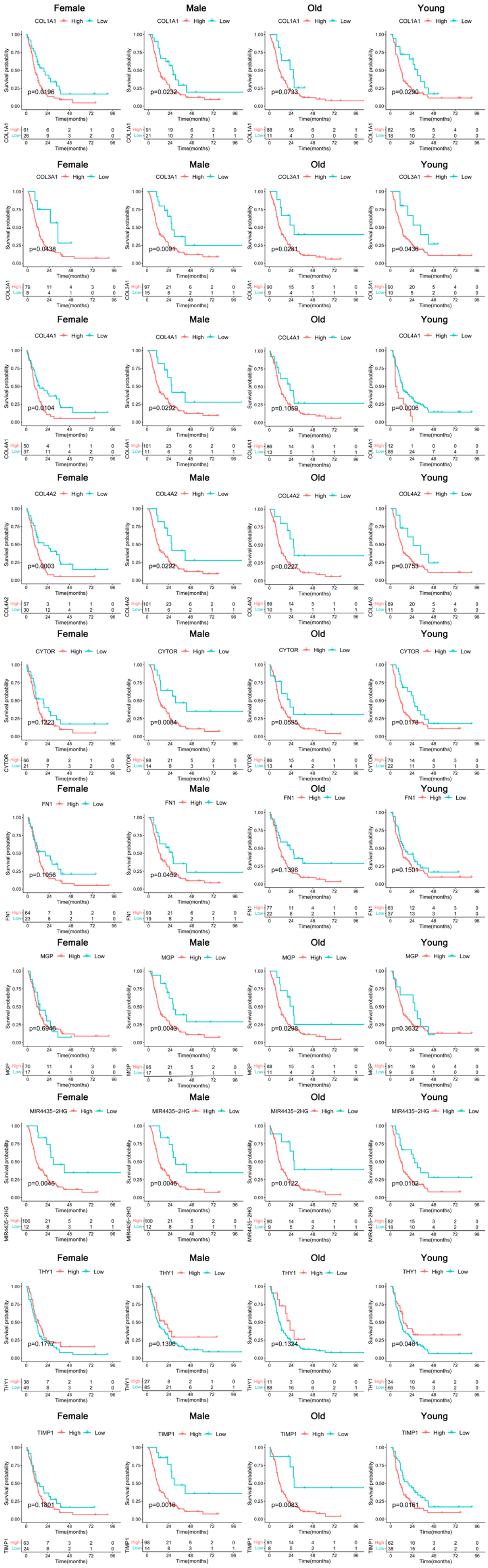
Female
Male
Old
Young
COL1A1 High Low
p=0.0196
p=0.0232
p=0.0733
p=0.0290
COL3A1 High Low
p=0.0438
p=0.0091
p=0.0261
p=0.0436
COL4A1 High Low
p=0.0104
p=0.0292
p=0.1059
p=0.0006
COL4A2 High Low
p=0.0003
p=0.0292
p=0.0227
p=0.0753
CYTOR High Low
p=0.1223
p=0.0084
p=0.0595
p=0.0178
FN1 High Low
p=0.1056
p=0.0452
p=0.1398
p=0.1501
MGP High Low
p=0.6948
p=0.0043
p=0.0298
p=0.3632
MIR4435−2HG High Low
p=0.0045
p=0.0045
p=0.0122
p=0.0102
THY1 High Low
p=0.1777
p=0.1396
p=0.1324
p=0.0461
TIMP1 High Low
p=0.1801
p=0.0016
p=0.0083
p=0.0161
Survival probability
Time(months)

Figure S7 Stratified Kaplan–Meier curve of top 10 DEGs between the high and low expression group in the CGGA GBM cohort.

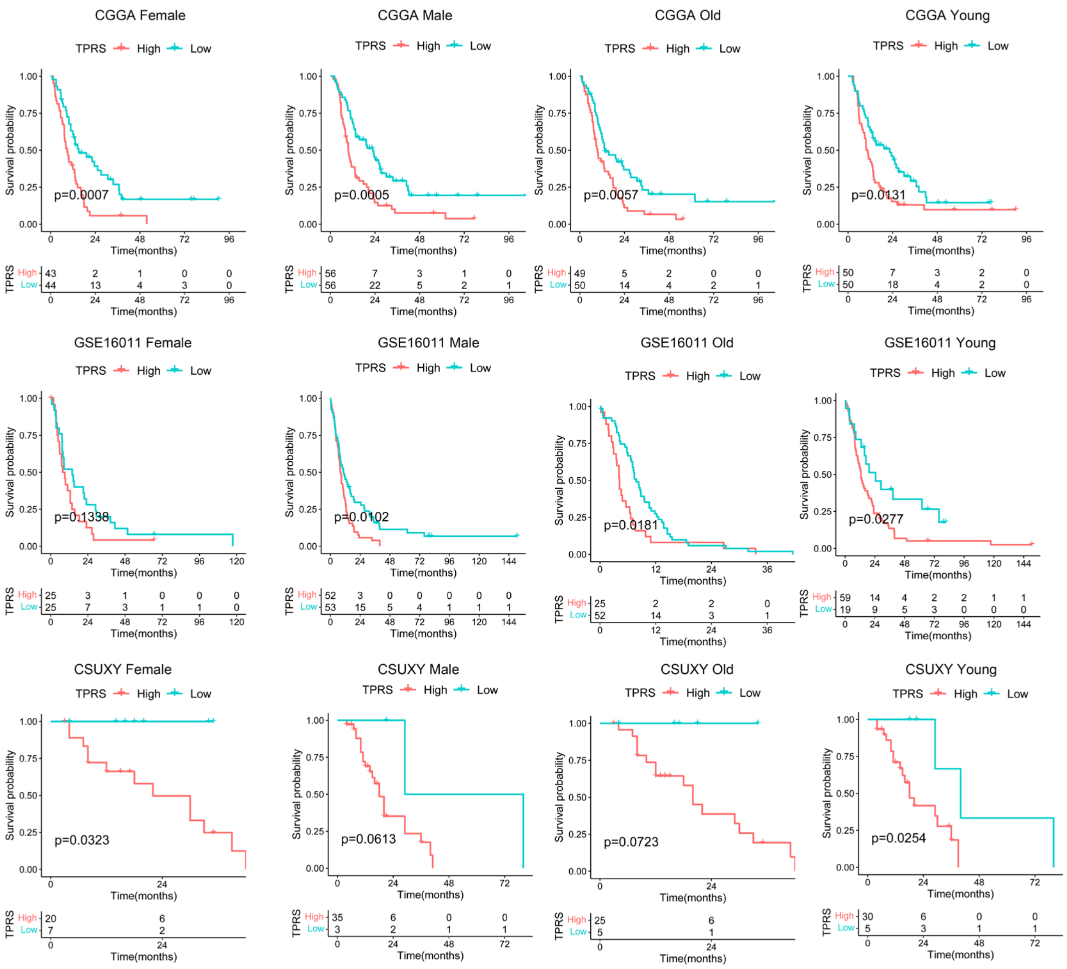

Figure S8 Stratified Kaplan–Meier curve of the TPRS high/low group in four GBM cohorts.

Supplementary Table 1.

Clinical information of specimens selected to perform scRNA-seq.

Sample	Age	Sex	GFAP	Ki67	P53	IDH1	Olig2	EGFR	MGMT
G3	66	M	+	15%+	wt	-	+	+	
G4	64	M	+	30%+	wt	-	+	-	+
G14	65	F	+	30%+	wt	-	+	-	
G15	69	F	+	25%+	wt	-	+	2+	
G17	73	M	+	10%+	+	-	+	1+	
G18	72	M	+	70%+	+	-	+	1+	
G19	56	F	+	40%+	wt	-	+	-	+
G21	46	M	+	30%+	wt	-	-	+	+
G22	67	M	+	10%+	+	-	+	+	-
